# Robust multiple frequency multiple power localization schemes in the presence of multiple jamming attacks

**DOI:** 10.1371/journal.pone.0177326

**Published:** 2017-05-11

**Authors:** Ahmed Abdulqader Hussein, Chee Yen Leow, Tharek A. Rahman

**Affiliations:** 1 Wireless Communication Centre (WCC), Faculty of Electrical Engineering, Universiti Teknologi Malaysia, UTM Skudai, Johor 81310, Malaysia; 2 University of Technology, Baghdad-10066, Iraq; University of Texas at San Antonio, UNITED STATES

## Abstract

Localization of the wireless sensor network is a vital area acquiring an impressive research concern and called upon to expand more with the rising of its applications. As localization is gaining prominence in wireless sensor network, it is vulnerable to jamming attacks. Jamming attacks disrupt communication opportunity among the sender and receiver and deeply impact the localization process, leading to a huge error of the estimated sensor node position. Therefore, detection and elimination of jamming influence are absolutely indispensable. Range-based techniques especially Received Signal Strength (RSS) is facing severe impact of these attacks. This paper proposes algorithms based on Combination Multiple Frequency Multiple Power Localization (C-MFMPL) and Step Function Multiple Frequency Multiple Power Localization (SF-MFMPL). The algorithms have been tested in the presence of multiple types of jamming attacks including capture and replay, random and constant jammers over a log normal shadow fading propagation model. In order to overcome the impact of random and constant jammers, the proposed method uses two sets of frequencies shared by the implemented anchor nodes to obtain the averaged RSS readings all over the transmitted frequencies successfully. In addition, three stages of filters have been used to cope with the replayed beacons caused by the capture and replay jammers. In this paper the localization performance of the proposed algorithms for the ideal case which is defined by without the existence of the jamming attack are compared with the case of jamming attacks. The main contribution of this paper is to achieve robust localization performance in the presence of multiple jamming attacks under log normal shadow fading environment with a different simulation conditions and scenarios.

## Introduction

Wireless sensor network localization can be achieved using techniques like received signal strength (RSS), time of arrival (TOA) and angle of arrival (AOA). The vast majority of these techniques are built on the physical properties of the wireless system design [1]. Following the widespread deployment of the location information services, there is an emerging event of the malicious attacks on the localization services. A large portion of these attacks are expected to affect the localization procedures so that the applications associated with the location information services will be extremely influenced. Location infrastructure is vulnerable to several attacks such as classical and physical attacks [2, 3].

The result of jamming attacks causes a huge deviation on the estimation of the sensor node position which leads to severe estimation error. Applications based on localization are directly affected as a result of these errors. Jamming attacks can inserting a false signal into the network due to the shared infrastructure medium of the wireless channel communication. For instance, a jammer can transmit a certain signal in order to outface the communications among the transmitter and receiver. The essential objective of the jamming attacks is to interfere with the wireless channel with an appropriate and powerful signals. Localization approach using range based strategy such as RSSI is extremely influenced by these attacks [3]. Meanwhile, localization of wireless sensor networks is facing a major issue due to the jamming attacks’ impact. There are considerable challenges and difficulties that ought to be researched on by researchers to cope with these dangerous attacks.

The task of security provision in wireless sensor networks is very demanding and includes several challenges such as protection against dangers, losses, damages, and attacks. The challenge becomes more complex given the limited input and output (I/O) resources, memory and computational power possessed by wireless sensor nodes. Avoidance of intrusion and upholding secrecy is among the most common security issues encountered in a wireless sensor network. The task of security of the access in wireless networks is very ambiguous against the wired or fixed networks. The reason for this is explained by the utilization of the wireless transmission medium of wireless networks. Access security of wireless sensor networks poses more challenges when compared to their traditional wired counterparts. This can be attributed to the hostile environment resource limitation of the sensor nodes. Wireless sensor networks are typically heterogeneous in nature. They consist of tiny sensors and actuators that make up their general-purpose computing elements. The computing elements are usually characterized by restricted resources such as bandwidth, computational power, peripherals and power, thus making it challenging and taxing to design a secure wireless sensor network system. Existing security schemes consume more energy and need large resources such as computational power, memory, etc. Other challenges such as network density and size, physical attack risks, nature and features of wireless communication channels, unknown topology before deployment etc. further complicate the provisioning of wireless security networks [4].

Wireless sensor networks have also attracted the attention of cryptographers and other security researchers [5–9]. Many recent works are incapable to achieve an exceptionally secured localization algorithm without performing the cryptographic strategies which impose additional hardware and computation requirement. The proposed method of this paper takes into account the robust localization performance of wireless sensor network in the presence of multiple jamming attack without the need of cryptographic techniques which indeed increasing the hardware complexity and the computational requirements. However, non cryptographic technique which implemented to cope with the presence of jamming attacks in the wireless network through detecting the jammers as well as eliminating its impacts remains unexplored. Hence, this paper proposes algorithms based on multiple frequency multiple power localization (MFMPL) technique to cope with the multiple types of jamming attacks under the shadow fading propagation model without utilizing cryptographic strategies. The promising performance of the new proposed algorithm is accomplished to attain accurate and powerful localization performance.

## Background

**Fig 1:**
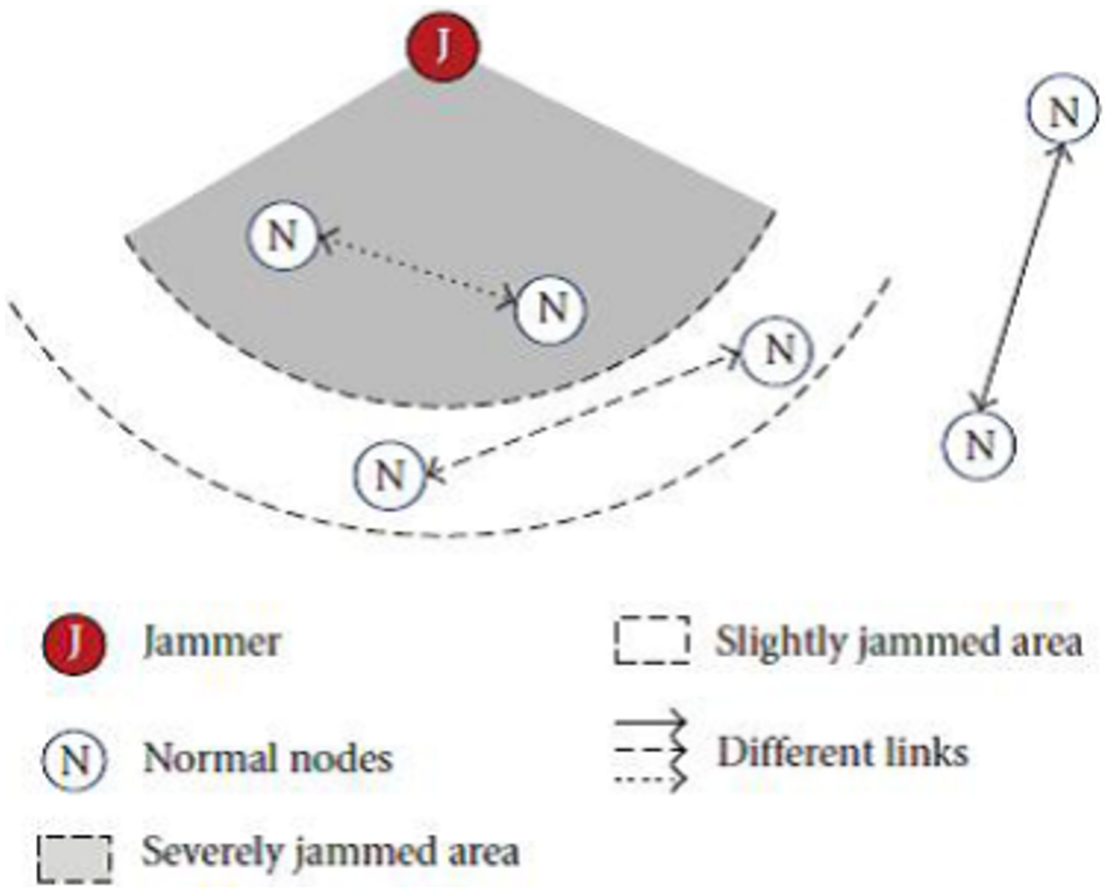
Illustration of varying jamming effects on different nodes.

Due to the broadcast nature of wireless transmission, the wireless signal is susceptible to other interferring signals if a proper frequency matching has occurred between these signals. Malicious node can eavesdrop the transmitted signal as well as injecting a false signal into the wireless network system. Jamming or man-made interference is the most famous kind of the physical layer attacks in wireless sensor networks to interfere, distort or distrupt the communication between the transmitter and the receiver. Along these lines, such attacker can easily generate a signal and cope with the targeted signal which leads to disrupt the communication. The main pivotal task of the physical layer in the wireless sensor network is responsible for the generation of the signal frequency as well as the selection of modulation, signal detection and authentication of the information [10]. Concurrently, jamming attacks have full access capability due to the shared infrastructure medium. Hence, it is clearly unsafe to deploy a sensor nodes in a such harsh and untrustworthy environment [11, 12].Fig 1 illustrates the sensor nodes deployment in the jamming area that examines various impacts of jamming attacks [13].

### Jamming attacks strategies

Jamming attacks are typical kind of the physical layer attacks in the wireless sensor networks. It is capable to disrupt the communication opportunity so that the essential aim of the jamming attacks is to inject a powerful signal into the channel in order to occupy it and prevent the direct sensor nodes communication. Stand on jamming attacks techniques, jammers can be categorized into various kinds as follows [14–16]:

- Constant Jammer: This type of jammers is constantly transmitting a radio signal into the channel.
- Deceptive Jammer: This kind of jammers injects continuous packets into the channel with no gap between the transmitted packets. As a result, normal communication will be tricked and trusting that it is a proper packet. Therefore, this jammer deceives the target receiver to keep accepting these inappropriate packets.
- Random Jammer: This kind of jammers constantly stands in between the jamming and the sleep action. The processing of this jammer is to infuse a signal into the channel for a specific time tj, then the sleep action will be started by switch off its signal for a specific time ts.
- Reactive Jammer: This type of jammer stay silent until an activity of the channel is detected and then it will start transmitting its packets.
- Capture and Replay Attack: A replay attack occurs when information is stored without authorization as a result of a security breach or eavesdropping and maliciously retransmitted to deceive the receiver to perform unauthorized operations like fake authentication, identification, or to duplicate a transaction. This is usually achieved when an attacker captures the login credentials of an authorized user as he attempts to login to a network and afterwards replayed. Replay attack may employ an effective and simple strategy called the denial of service (DoS) for locally heard data packets. These packets are then passed by another forwarding node, leading to an increase in the congestion level and redundancy at each forwarding node. A replay attack can be carried out anytime and anywhere within the network.

### Fundamentals of the range-based received signal strength indicator technique

The basic principles of the RSS ranging portrays the relationship of the wireless signal power between the transmitter and the receiver according to the distance among them. This relationship can be described through Eq (1).

$$\begin{array}{c} P_{r}=P_{t}*{\left(\frac{1}{d}\right)}^{\alpha } \end{array}$$ (1)

where

Pr: the wireless signal received power.

Pt: the wireless signal transmitted power.

d: the distance between the sending and receiving nodes.

*α*: the transmission parameter value depends on the propagation environment.

By converting Eq (1) to dB, then

$$\begin{array}{c} 10\log P_{r}=10\log P_{t}-10\alpha \log d \end{array}$$ (2)

Eq (2) can be rewritten as:

$$\begin{array}{c} P_{r}\left(dB\right)=A-10\alpha \log d \end{array}$$ (3)

From Eq (3), the parameter A and *α* indicate the relationship between the received signal strength and the distance for a such transmission path.

Currently, the channel propagation models are commonly utilized in wireless sensor networks which include free space and log normal shadow fading environment. In fact, the log normal shadow fading is more prevalent and suitable model for indoor as well as outdoor localization. Besides, it has been considered to be the most compatible for a real channel design of the wireless sensor network localization, because of its flexibility to various environmental configurations. On the other side, the free space model has another merit: (1) Free space model is more extensive transmission distance as compared with the carrier wavelength and the antenna size as well. (2) The line of sight transmission has been considered in this model, so that it does not have the obstruction issue between the transmitter and the receiver [17, 18].

Assume that the sensor node is deployed at a distance d in the network so that the received signal power can be obtained accordance to Eqs (4) and (5).

$$\begin{array}{c} P_{r}\left(d\right)=\frac{P_{t}G_{t}G_{r}\lambda^{2}}{{\left(4\pi \right)}^{2}d^{2}L} \end{array}$$ (4)

$$\begin{array}{c} PL\left(dB\right)=10\log\frac{P_{t}}{P_{r}}=-10\log\left[\frac{\lambda^{2}}{{\left(4\pi \right)}^{2}d^{2}}\right] \end{array}$$ (5)

In Eq (4), PL(dB) is the power path loss in dB, *G*_*t*_ and *G*_*r*_ are the antenna gain, and L is the system loss parameter, which is not related to the transmission loss. *G*_*r*_ = 1, *G*_*t*_ = 1 and L = 1 are ordinarily take these values for the system design. Eq (5) describes the formula of the signal attenuation utilizing logarithmic form.

The most common propagation model which is appropriate for both of indoor and outdoor localization as well as confers several parameters which can be implemented for various environment is the log normal shadow fading model. The mathematical expression for the log-normal shadowing model can be obtained according to Eq (6).

$$\begin{array}{c} PL\left(d\right)\left(dB\right)=\bar{PL}\left(d\right)+X_{\sigma }=\bar{PL}\left(d_{o}\right)+10\log\alpha \left(\frac{d}{d_{o}}\right)+X_{\sigma } \end{array}$$ (6)

According to Eq (6) *d*_*o*_ is the reference distance, which relies on upon the empirical value, while *α* is the path loss exponent, which relies on upon the channel propagation medium and in case of obstructed environment its value becomes larger, the parameter *X*_*σ*_ is a zero-mean Gaussian random variable. The parameters *d*_*o*_, *α* and *σ* depict the path loss channel model for a particular distance between the transmitter and the receiver. The model can be implemented for a generic design and analysis of the wireless systems [19, 20]. Regarding the above two sorts of propagation models, log normal shadow fading is more realistic for wireless sensor network applications, due to the inclusive merit and the capability to be designed for such environments.

### The relationship of frequency and power for the path loss propagation model

The log-normal path loss between the transmitter and the receiver of the channel propagation model can be depicted according to Eq (7) [20, 21]:

$$\begin{array}{c} P\left(d\right)=P\left(d_{o}\right)-10\alpha \log\frac{d}{d_{o}}+X_{\sigma } \end{array}$$ (7)

where P(d) is the received power of the wireless sensor at a such distance d to the landmark (Anchor node), *P*(*d*_*o*_) is the power loss in a free space and is usually considered as one meter, *α* is the path loss exponent, while *X*_*σ*_ is the effect of the shadowing parameter with a variance of *X*_*σ*_, which can be expressed by Eq (8).

$$\begin{array}{c} X_{\sigma }∼N\left(0,\sigma^{2}\right) \end{array}$$ (8)

The power loss *P*(*d*_*o*_) is the most usually depends on the transmitted frequency which has been used. Thus, it is more often considered to be a transmission frequency dependent parameter [22]. Moreover, the path loss parameter describes the amount changing of the RSS measurements with respect to the distance, which inclines to increase its value in case of a higher transmission frequency used by a such device. Eq (9) demonstrates the relationship between the path loss exponent with respect to the utilized frequency transmission [23].

$$\begin{array}{c} \alpha \propto f_{\left({\mathrm{MH}}_{z}\right)} \end{array}$$ (9)

Where:

*α*: Path loss exponent.

*f*: Beacon signal frequency which is used by anchor node.

Finally, severe changes of the received signal power which is caused by the shadow fading impacts due to the obstruction environment that exists in its propagation paths. Therefore, the experimental measurements proved that the shadow fading is depended directly on the transmitted frequency and the power level as well [24].

The signal propagation model parameters related to such RSSI ranging techniques are generally determined by online or offline estimations. In fact, online measurements exhausted more computational time as well as the communication resources. Regardless, in practical situations, the signal propagation parameters are entirely hard to be measured [25]. Hence, the precise determination of the signal propagation parameters absolutely leads to a perfect measurement of the RSS rangings [26].

In perspective of the path loss propagation model, the probability density function of RSS measurements in a such landmark (Anchor node) which has been assembled from (N = 1, 2,………N) landmarks can be formed as appeared in Eq (10).

$$\begin{array}{c} f_{\left(x,y\right)}\left(P\right)=\prod_{i=1}^{N}\frac{10}{\log10\sqrt{2\pi }\sigma_{i}P_{i}}\exp\left[-\frac{P_{i}}{8}{\left(\log\frac{d_{i}^{2}}{\hat{d_{i}^{2}}}\right)}^{2}\right] \end{array}$$ (10)

where Pi = [p1…pN] refers to the received power vectors for N landmarks. Also,

$$\begin{array}{c} P_{i}={\left(\frac{10\alpha_{i}}{\sigma_{i}\log10}\right)}^{2} \end{array}$$ (11)

$$\begin{array}{c} \hat{d_{i}}=d_{o}{\left(\frac{P(d_{o})}{P_{i}}\right)}^{\frac{1}{\alpha_{i}}} \end{array}$$ (12)

$\hat{d_{i}}$ is the estimate of distance *d*_*i*_ to the *i*th landmark (Anchor node). (x, y) refers to the estimated position of the unknown sensor node which is computed from the RSS readings. In this way, the parameter Pi comprises of the path loss component as well as the shadow fading standard deviation parameter which is clearly depended on the transmitted frequency and power level for a such landmark.

## Related work

A few studies related to the localization of wireless sensor network which dealing with the vicinity of jamming attacks. The vast majority of the existing works are concentrating on localizing the jammers in the network. On the other side, another few works concentrating on finding the sensor node position under this harsh environment. A localizing of jammers techniques such as Centroide Localization (CL) and Weighted Centroide Localization (WCL) has been presented in [27]. CL algorithm assembles and averages the coordinates of the jammed nodes to determine the jammer location in the network. This algorithm suffers from the propagation irregularities with a high sensitivity to the node distribution in the network. Likewise, WCL is a strengthened form of CL approach with enhancement of the jammer localization accuracy. An improved technique known as Virtual Force Iterative Localization (VFIL) [27] uses an iterative method for estimating the jammer location. This method demonstrates a high accuracy as well as less sensitivity for the sensor node distribution in the network but it takes more computations than CL and WCL.

A reactive technique to detect and locate the jammer position through utilizing a distributed collection of wireless sensor nodes has been investigated in [28]. This work is using autonomic computing as a framework and employing a circular curve fitting algorithm. The determination of the approximate jammer position has been accomplished via analyzing the active nodes position and targeted nodes location as well. The effect of the medium access control (MAC) protocol on timing and collision was ignored in this work. In addition, the location of the sensor nodes has to be known a priori in order to estimate the position of the jammer in the network. Therefore, this work is focusing on localizing the position of the jammer in the network only without any solution for accurate localization of the sensor node in the presence of jamming attack environment.

The point of view which has been taken into consideration by these works is to get an appropriate action, alert the system network or it has been accomplished just to test the impacts of jammers on the localization performance. Our previous work [16] explores more details about localizing the jammers related to the wireless sensor networks.

A scheme to localize a wireless node using jamming attack as the advantage of the network has been proposed in [29]. This approach consists of two steps. In the first step, it discovers the location of the jammer using power adaptation technique and then uses these properties to localize the jammed nodes. But in this approach it is assumed that the sensor nodes have high transmission power which is not always the case for energy constrained sensor nodes.

An extensive study on the implementation and possibility of jamming attack detection of the wireless sensor networks has been carried out. One typical example of a jamming attack effect is a four different jamming attack models were proposed by [30] using various detection techniques to identify jamming attacks in sensor networks. They classified poor radio link using packet delivery ratio (PDR), before performing a consistency check to ascertain if jamming was the root cause. Although their method was quite effective in detecting jamming attacks, it could not offer counter or preventive measures against jamming.

Instead of utilization such fundamental statistical strategies, multimodal techniques, for example, joining PDR with signal strength measurements could deliver better performance. In an ordinary situation with no interference, a high signal strength coincides with a high PDR. While in the case of the signal strength is low, i.e. it is near to the noise level, the PDR value will likewise be low. On the other side, a low PDR does not neccessary infer a low signal strength reading, it might be that all neighbor nodes have a dead battery due to consuming power resources, device fault or the node is jammed. For the case of jamming, the signal strength ought to be high, which negates the low the PDR.

Regarding the relationship between the signal strength and the PDR ratio, an advanced jamming detection strategy has been investigated in [31]. The basic idea of this proposed approach is to measure the signal strength readings and the PDR ratios during the non -interfered network operation and construct these information in a table (PDR, SS). From this information, it can ascertain an upper bound for the maximum signal strength (SS) that would have introduced a specific PDR value in a non -jammed situation. According to this bound information, the (PDR, SS) plane is apportioned into legitimate and jammed regions. This work indicates that the (PDR, SS) values for all jammers unmistakably fall inside the jammed area which is recommending that regions classification is achievable. Therefore, PDR considered to be the most robust statistical strategy which can be used for differentiating the jamming attack from an overcrowded network situation and it is suitable for various jammer models as well. However, it is still unable to differentiate the jamming attacks caused by another network that outface the communication among the sender and the receiver.

A novel wormhole attack detection technique has been presented in [32]. The main contribution of this work is to detect wormhole attacks in a geographic routing protocol (DWGRP). The detection of malicious nodes is achieved through selecting the best and the most reliable neighbors based on pairwise key pre-distribution technique and the beacon packet as well. Hence, this work depends directly on identifying trust neighbors with respect to the secure shared keys which require extra complex hardware. Furthermore, this method will fail in the presence of non-cryptographic attacks and susceptible to a cryptographic attacks as well.

A new Single Stage Memory Random Walk technique and Single Stage Memory Random Walk with Network Division (SSRWND) have been proposed in [33]. This work has been implemented in order to detect node replication attacks in wireless sensor networks. The main contribution of this work aims to reduce the communication and memory costs while keeping the detection probability high. This work depends directly on the witness nodes in the network in order to get more effective and robust clone detection. Hence, the requirements of this work include uniform distribution and non-deterministically with equal probability of the witness nodes should be selected. Furthermore, it is assumed that static sensor nodes knowing their locations as well. The assumptions of this work are impractical and still susceptible to cryptographic attacks and the capture and replay attacks.

Given the graveness of the effects of radio jamming attacks, a number of studies have also proposed some anti-jamming techniques. In [34], the effect of radio jamming can be neutralized if the power of transmission nodes is proportional to the power of the jamming radio. The authors assumed that the jamming effect on source-receiver communications is not isotropic. They improved the transmission power of a testbed with Mica2 motes and used it to study the jamming effect. Therefore, the proposed method takes into consideration the effects of jamming attacks on the transmission power without any solutions to cope with jamming.

The technique presented in [35] uses an optimization technique to address the jamming problem. The authors represented the jamming issue as an optimization problem and tried to solve it to determine the probability of transmitting a radio signal from both the network and jammer’s end, in order to achieve optimal jamming and defense effectiveness. The focus of their research was to understand the interactions that take place between the jammer and the nodes in a network only.

Regarding to the scheme in [36] which studied insider jamming attacks and found that jamming attacks can be carried out by an attacker once a compromised node is exploited to reveal common cryptographic information. To address the insider jamming attacks challenge, a compromise-resilient anti-jamming scheme was proposed. The scheme uses group keys that are shared by sensor nodes to determine the physical channel used by a sensor network. This method needs to generate a new key group of cryptographic information which will be utilized for all the non–compromised sensor nodes in order to cope with the single insider jammer. This method will fail if multiple jammers exist as well as it needs extra hardware which increasing the complexity of the wireless sensor network design.

The channel surfing technique proposed in [37, 38] to address the jamming interference. The authors studied two methods of channel surfing: coordinated channel switching, where the radio channel of the entire sensor network is changing dynamically, and the spectral multiplexing, where radio channel is changed by the nodes in the jammed area with the boundary nodes acting as relays. This technique is a complicated strategy to cope with the jamming attacks as well as taking more time to repair the sensor node communication and it will fail in the presence of multiple jammers in the networks.

An approach based on frequency hopping spread spectrum (FHSS) and direct sequence spread spectrum (DSSS) has been presented in [39] which is considered to be the best countermeasure to cope with the jamming attacks. The basic strategy of this approach is that the jammed nodes utilizing the channel diversity to establish the communication with the sensor nodes which are outside the jamming area. However, this strategy is only implemented for the routing process and the localization of the sensor node is not taken into consideration in this method.

Although traditional methods of cryptography, e.g. authentication can be used to protect against conventional attacks from adversaries such as false message injections, non-cryptographic attacks are, however, completely orthogonal and have the ability to corrupt the signal strength measurement processes. Unfortunately, traditional security services are incapable of militating non-cryptographic attacks, thus the need to investigate the impact of these attacks on localization algorithms with the aim of proposing methods that can be effectively used to detect and eliminate such threats from the network. Despite advances in the domain of secure localization [40–43], there is yet to be any study in the area of robustness of RSS-based localization algorithm generation against physical attacks. The evaluative study in this paper is therefore significant in its contribution to wireless sensor network designs, particularly in the area of protocol decisions drivers, which will enable engineers to make better informed decisions about whether there is a need for a more complicated security localization algorithm.

The network availability attacks are usually indicated as a Denial of Service (DoS) attacks, which going to make the wireless network unavailable, corruption or disorder the network performance. Usually it can target one of each layer of the open system interconnection (OSI) layer of the sensor network. The optimized attack schedule has been investigated in order to streamline the effect on the system performance due to the insufficiency of energy at the attacker side. An optimal DoS attack that maximizes the Linear Quadratic Gaussian (LQG) control cost function under the energy constraint has been introduced in [44]. The work start with analyzing the properties of the cost function under an arbitrary attack schedule, then deriving the optimal jamming attack schedule and the corresponding cost function. System stability under this optimal attack schedule is also considered. In this work, different examples are provided to demonstrate the effectiveness of the proposed optimal denial-of-service attack schedule. This work considers the effectiveness of the proposed optimal jamming attack policy in WNCs, but it does not take into account the impact of jamming attacks on the localization accuracy.

Optimal attack schedules to maximize the expected average estimation error at the remote estimator have been demonstrated in [45]. This work provides the optimal attack schedules when a special intrusion detection system (IDS) at the estimator is given. The work investigates how an attacker should schedule its Denial of Service (DoS) attacks to degrade the system performance. Specifically, it considers the scenario where a sensor sends its data to a remote estimator through a wireless channel, while an energy-constrained attacker decides whether to jam the channel at each sampling time. In this work, a single sensor scenario has been studied and the impact of jamming attacks on the localization accuracy does not take into consideration.

## Network and adversary model

For the network model, N sensor nodes are deployed in a non authoritative region which has an obligation to estimate their positions with respect to a range distance measurements for a number of implemented anchor nodes / access points (APs) in the network. The anchor nodes (APs) are deployed around the perimeter of the network with known locations a priori. Anchor nodes transmit beacon signals according to a multiple frequency and power strategy with their coordinates denoted as (x, y). At the sensor node, reference position (x, y, d’) is constructed from the received beacon signals, d’ refers to the range distance estimation between the sensor node and each anchor node in the network. The range distance estimation is obtained as a result of measuring the received signal strength (RSS). When required valid beacons are available with their reference position, sensor nodes can applying lateration and trilateration techniques in order to estimate the sensor node location in the network.

The adversary is capable to perform straightforward signal manipulations in the wireless sensor networks. For instance, creating a fabricated signal, signal cancellation, signal modification and re-requesting of signal sequences. There are established security concepts such as passive or active attackers. Besides, there are a characterization of other notions that are more particular to wireless sensor network such as mote-class or laptop-class and the start of the attacks either inside or outside security border. Along these lines, definitions and further information of adversaries related to the wireless sensor networks can be found in [33, 46–49]

The shared infrastructure of the transmission medium in the wireless sensor networks confers the chance for an adversary to dispatch wireless physical jamming attacks. To give a straightforward illustration, a malicious node can ceaselessly transmit a radio signal with a specific end goal to hinder any valid access to the medium and/or interfere with reception. This demonstration is called jamming and the malicious nodes are known as jammers.

**Fig 2:**
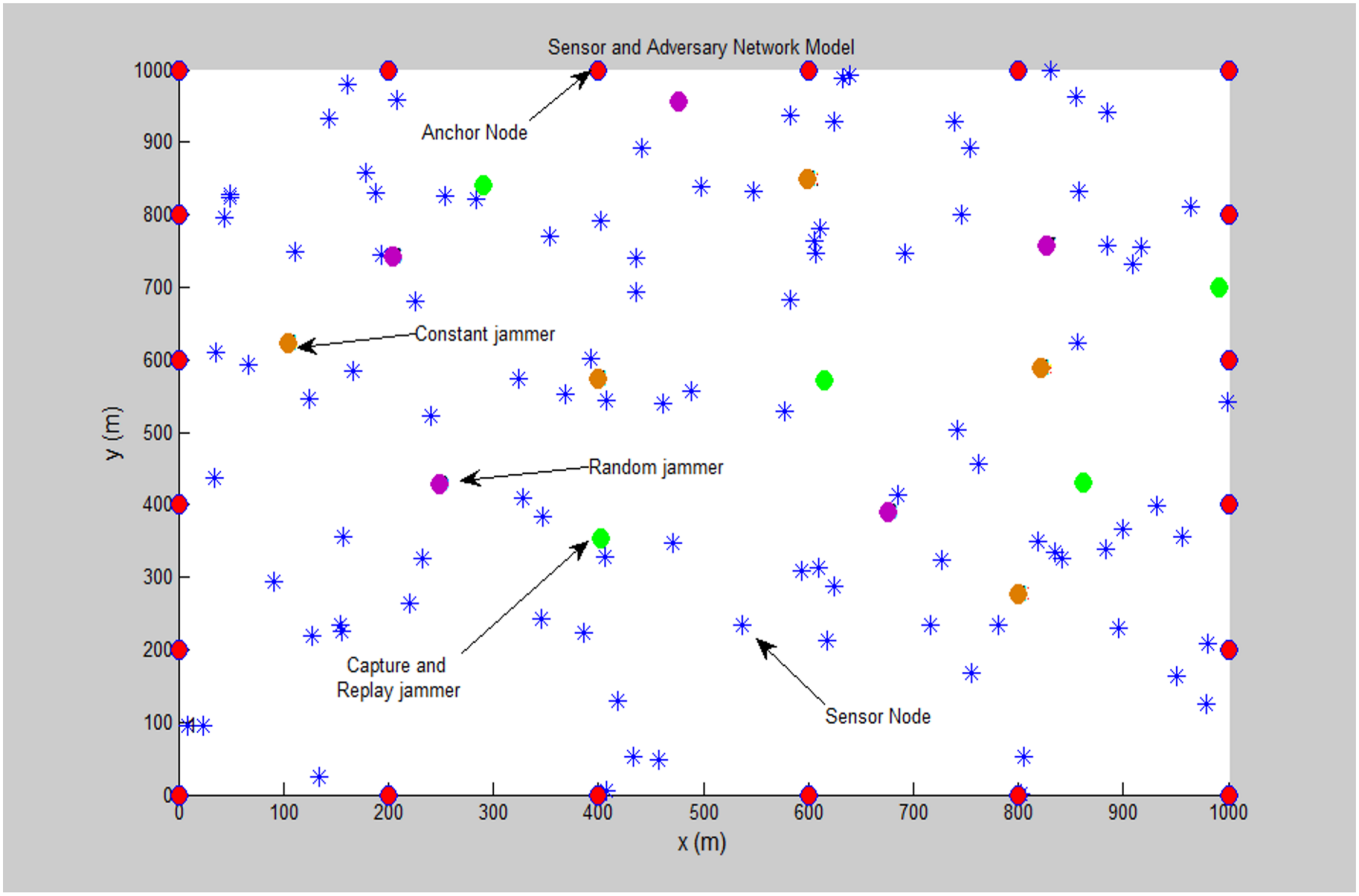
A network model with anchors, sensors and multiple jammers.

For the adversary model, there are multiple jammers placed randomly in the network. Constant and random jammers interfere the wireless sensor network channel via two fixed frequencies. On the other hand, all the transmitted beacon signals will be captured and replayed by the capture and replay jammer. The coverage area which is occupied by each jammer node can be described as a circle centred at the point of the jammer location in the network. Moreover, through applying a powerful antenna, transmission range for each jammer is wider and stronger than the range of the sensor nodes. Fig 2 shows a network scenario with anchor nodes (APs), sensors and the jamming sensors.

In order to simulate a powerful and effective jamming attacks in the network system, multiple jammers indicated by three different types have been implemented as follows:

- Constant jammer.
- Random jammer.
- Capture and replay jammer.

Constant and random jammers work in a different frequency ranges denoted by 400 and 600 MHz respectively. These jammers will target the anchor node transmitted beacon signals at these frequencies only. The anchor node transmitted beacon signals will be captured by the capture and replay jammers and retransmitted in the network. Therefore, taking these jammed beacons into consideration in the localization process will lead to a huge estimation error for the sensor node position due to the deviation of the measured received signal strength (RSS). This kind of signal strength attack detrimental to the whole wireless sensor network localization process. Hence, the impact of this considerable attack should be ceased.

## Pre-localization mechanism of filtering beacon signals

Despite the high availability of the received beacon signals which is accomplished through a powerful technique of the proposed algorithm in order to cope with the constant and random jammers in the network, these available beacons can not be considered to be valid due to the impact of the capture and replay jammers. Hence, each sensor will receive beacons from different anchor nodes (APs). Since the beacon signals are sending two different sets of frequencies shared and distributed through a uniform sequence between the implemented anchor nodes as well as jammers cannot attack all the frequencies at once, sensor nodes will receive beacons even in spite of presence of constant and random jammers. The only problem now is with the capture and replay jammer which directly affect the localization performance through interfering and injecting invalid beacons through the wireless sensor network.

**Fig 3:**
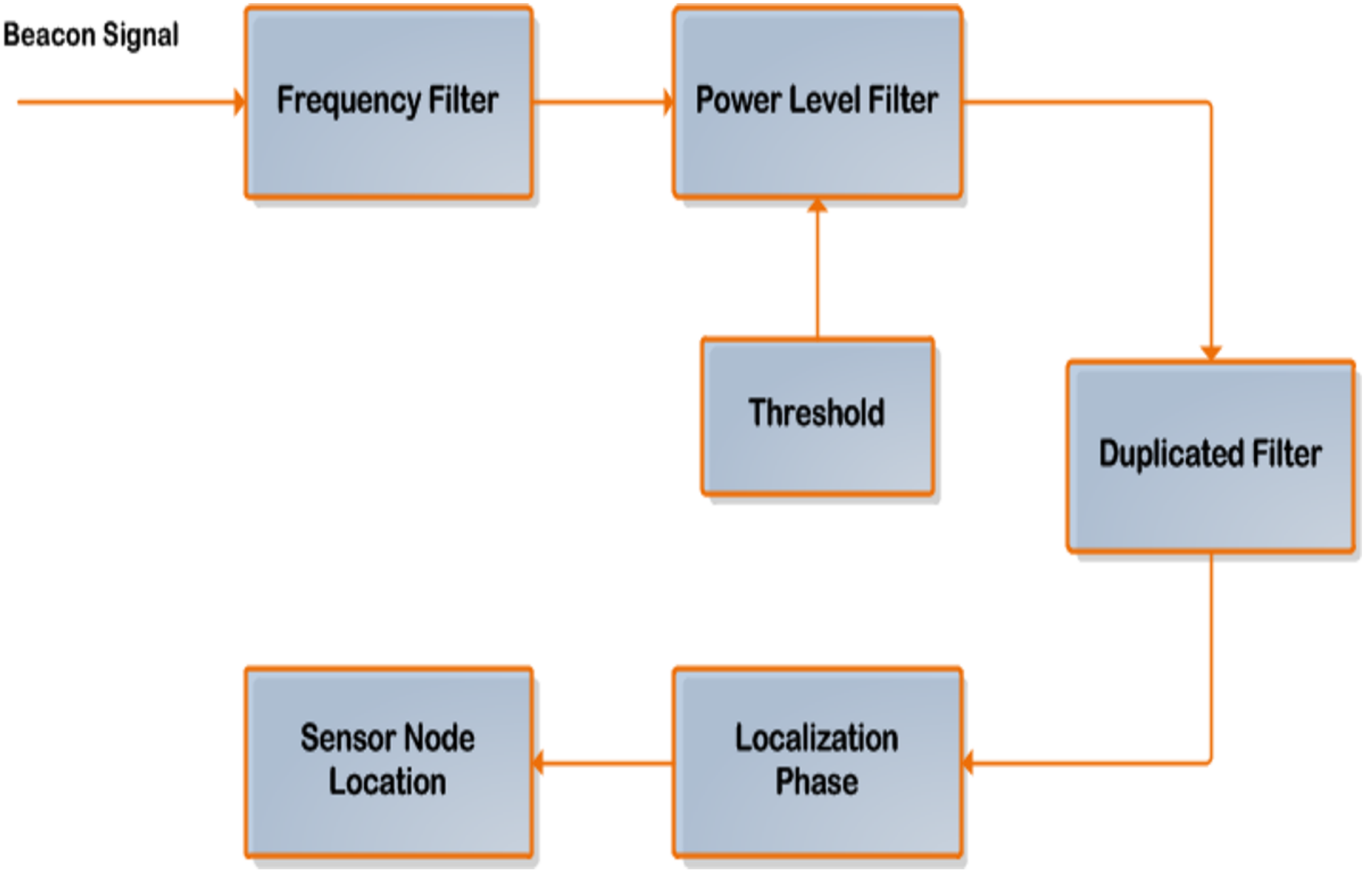
Proposed beacon signals filter technique.

Valid beacons from the access points will be captured and replayed by the jammer. This will influence the distance calculations using RSS ranging and the effect is cascaded to the sensor node location estimation. For this problem, set of filtering techniques at the sensor nodes have been proposed. Whenever the sensor node receives the beacons, it will be forwarded consistently through these filters to drop the replayed beacons. Fig 3 displays the block diagram of the filtering stages.

- Frequency Filter: This filter will estimate the frequency of transmission for the access point and match it with the frequency of the received beacon from the access point. According to Eq (13), if there is any deviation in the estimated received frequency with respect to the actual transmitted frequency it will be dropped and does not take into consideration of the localization process.
  $$\begin{array}{c} ESS=EstimatedFrequency-ActualFrequency \end{array}$$ (13)
  Non zero ESS value indicates an error and the beacon will be dropped.
- Power Level Filter: When the frequency validated beacons comes to Power level filter, the received signal strength is calculated and the packet is buffered. For the subsequent beacon from the access point, the power level is checked for lying in proportion with respect to the power level. The beacons not in proportion to the power level are just dropped and one packet passing the power level test is given to the duplicate filter. Ordinarily, the threshold value ought to be used to settle on an appropriate choice indication at the power level filter stage, as well as it is considered to be an important criterion for the detection process. Therefore, for each beacon signal passed through this filter if the error in the RSS measurements received from a certain anchor (AP) with a percentage error exceeds 15% of the actual RSS ought to be neglected, while RSS error less than 15% ought to be accepted and this beacon forwarded to the last filter stage.
- Duplicated Filter: If more than one valid beacon having the same frequency and power is received from the same anchor node, then only one is kept while the others will be dropped. The decision strategy of the beacon selection which has been accepted is based on comparing the RSS error for each received beacon with respect to the threshold value indication. Therefore, the beacon with a minimum RSS error will be accepted and passed to the localization stage.
- Localization: Using the valid beacons from different anchor nodes, the localization module will extract the coordinates of the anchor points from these valid beacons and estimate the optimized distance between the sensor node with respect to each anchor points using the averaging RSS values based on the proposed algorithms which have been achieved for the network design. Regarding to this information, lateration and trilateration techniques are applied in order to determine the sensor nodes position.

**Fig 4:**
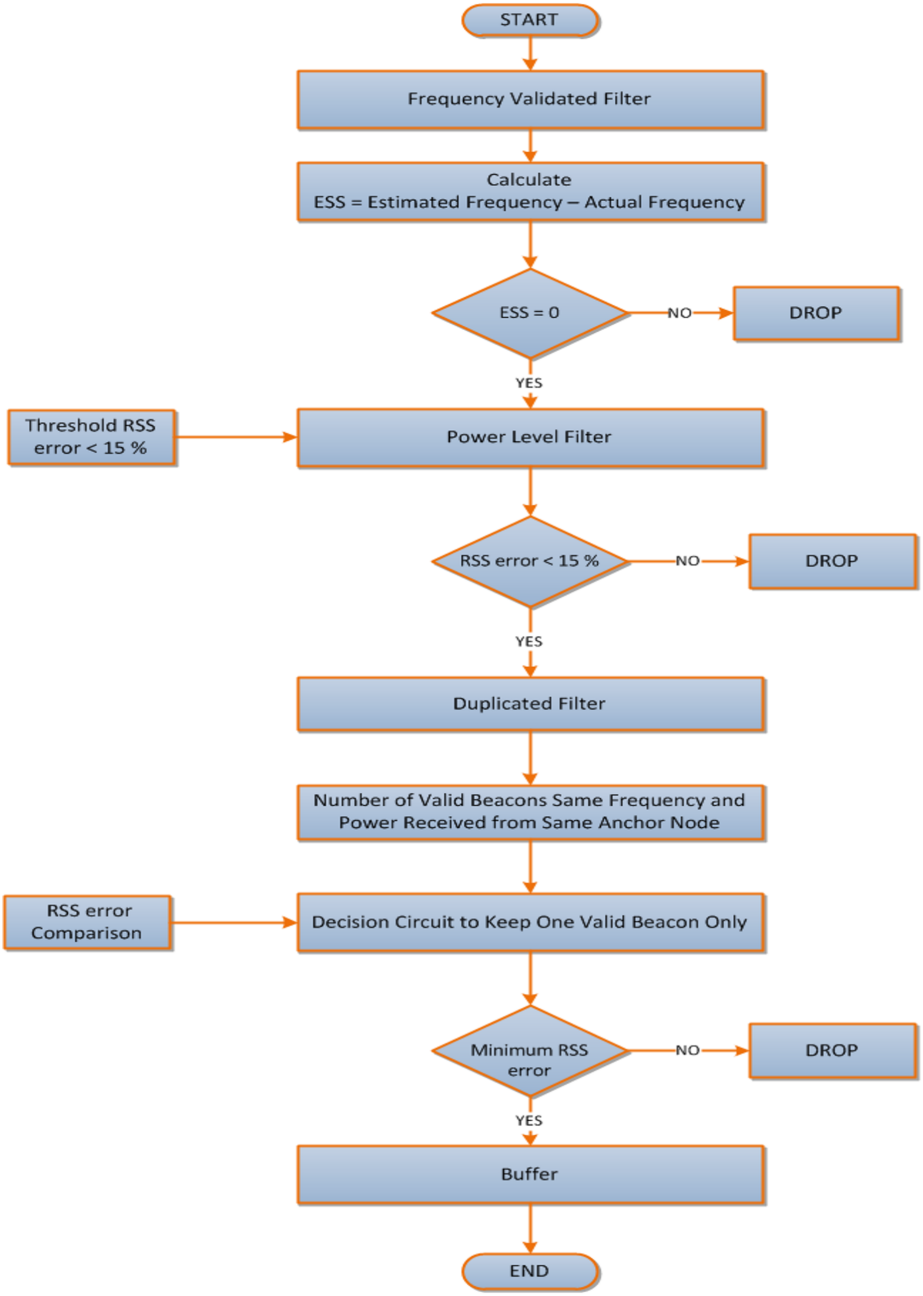
Analytical flow chart of the pre-localization mechanism for filtering the beacon signals.

The analytical flow chart which depicts the strategy of the pre-localization stage for filtering the beacon signals of the proposed solution can be indicated according to Fig 4.

In order to accomplish the robust localization accuracy in the presence of multiple jamming attacks environment which is considered to be the main objective of this paper, the proposed solutions have been implemented.

## Proposed solution

The proposed algorithm is a function of multiple frequency multiple power transmission achieved by two techniques: a Step Function Multiple Frequency Multiple -Power Localization (SFMFMPL) and a Combination Multiple Frequency Multiple Power Localization (C-MFMPL). Keeping in mind the end goal is to relieve the impacts of shadow fading, the averaging technique for all the RSS readings with respect to the transmitted frequencies has been also accomplished. In our proposed approach, the sensor nodes have the prior knowledge about the expected sequence of the frequency and the power level for the transmitted signals at each anchor / access point (AP). The proposed scheme can address the effect of the constant and random jamming attacks since these two types of jamming sensors can broadcast in only one frequency and will jam the packets sent at that frequency. However, the main impact of these jammers will appear for achieving a robust RSS averaging technique which is implemented to deal with the shadow fading effects. Hence, the new strategy of our proposed solution is accomplished through utilizing two sets of multiple frequency transmission shared between the deployed anchor nodes as well as distributed in a uniform sequence. With an aim to bypass the interference impact caused by capture and reply jamming attack which is considered to be the most dangerous type of jamming attacks, a proper technique has been proposed to filter out those invalid beacons by using three stages of filters. Therefore the received beacons will be checked by the frequency filter, power level filter and duplicated filter to remove the replayed beacons and applying the lateration using nonlinear least squares as well as trilateration techniques to estimate sensor node position.

It is assumed that the sensors and the anchor nodes are precisely timed synchronized. This is possible through utilizing a quartz crystal clock which is implemented for all sensors and anchor nodes. With the assistance of this method all of them are time synchronized in the network as well as it is simple and cost effective to be achieved [50, 51]. Time synchronization can be accomplished through another several techniques suitable for wireless sensor networks. Further information related to time synchronisation has been presented in [52].

### Robust multiple frequency multiple power localization (MFMPL) approach

**Fig 5:**
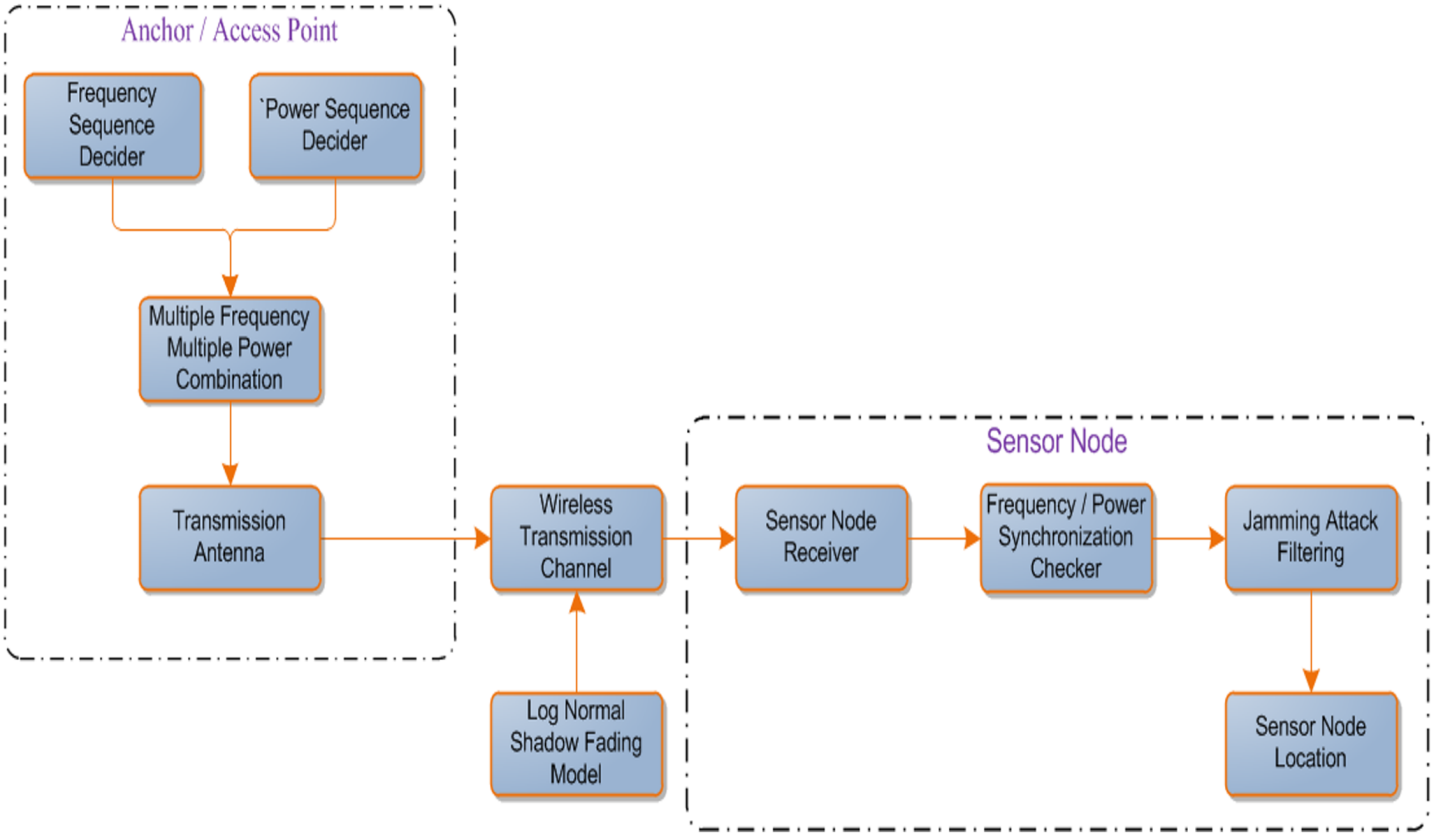
The functional block diagram of the C-MFMPL technique.

Multiple frequency and multiple power transmission have been performed in order to determine the sensor node location in the network. Definitely each sensor node has a prior knowledge about the specific transmitted frequency and the power level that used by the deployed anchor nodes in the network as well as the next decided frequency and power level sequence. At the sensor node side, the receiver attempts to synchronize the prospect frequency and power level as well as checking whether any interference existence in its path due to jamming attacks. The estimation of the sensor node location has been obtained through applying the lateration as well as trilateration technique. Furthermore, to avoid receiving invalid beacons at each sensor node due to capture and replay jamming attacks, a set of filters are used. The functional block diagram of our previous work (SF-MFMPL) can be found in [53]. Fig 5 outlines the functional block diagram of the (C-MFMPL) scheme.

The proposed strategy which is specified by multiple frequency multiple power localization (MFMPL) can be achieved via two phases as follow:

### Initialization phase

Regarding the initialization stage, every anchor node will pick a random seed and utilizing it to outline the frequency estimation function as appeared in Eq (14).

$$\begin{array}{c} Freq\left(t\right)=F(t,S_{t}\left(seed\right)) \end{array}$$ (14)

Frequency calculation function is determined by this random seed at the current time. The beacon signal which be transmitted at that time is denoted to the frequency calculation function. St refers to the seed generation function which creates the seed for a specific time with respect to the present time and the initial seed value. F and St refer to the initial seed for the beacon signal transmission by each anchor node which is kept in a protected memory of the sensor node with the goal that it is very hard by an attacker to estimates these values. In addition, anchor nodes transmit beacon signals with a multiple power levels in a consecutive way. Therefore, t power level sequence should be well known in priori by each deployed sensor node in the network.

According to the proposed technique based on SF-MFMPL, each AP (access point) is specified by a function of frequency and power level in a step wise over the possible values of transmission. The step function within the step duration is kept different for different anchor nodes (APs). A sample of a step function can be described by the Eq (15). Further details which describe the step function mechanism of the proposed strategy SF-MFMPL can be found in our previos work [53].

$$\begin{array}{c} f\left(t\right)=\{\begin{array}{cc} a & 0\leq t⩽3T\\ b & 3T<t⩽7T\\ c & 7T<t\leq 8T \end{array}\} \end{array}$$ (15)

A step function multiple frequency multiple power localization (SF-MFMPL) approach which is used in the simulation test to relieve the impacts of log normal shadow fading. The mathematical representation of the averaging operation for N (RSS) values gathered from a certain anchor nodes has been implemented by the proposed solution in accordance with Eqs (16) and (17) respectively.

$$\begin{array}{c} F=\{f_{1}\left(p_{1}\right),f_{2}\left(p_{2}\right),f_{3}\left(p_{3}\right)\} \end{array}$$ (16)

$$\begin{array}{c} averageRSSI_{i}=\frac{1}{N}\sum_{j=1}^{N}RSSI_{ij} \end{array}$$ (17)

The other proposed method in this study which has been utilized to achieve the multiple frequency and power level transmission mechanism can be identified by a combination of all the decided frequencies and power levels used for the beacon signals transmission via each anchor node in the network. However, the proposed method based on Combination Multiple Frequency Multiple Power Localization (C-MFMPL) has been built to face the log normal shadow fading model which obtains the essential features of the multiple frequency and power level as well as the averaging technique of all the received signal strength all over the transmitted frequencies in facing and overcoming the shadow fading impacts.

Hence, the mathematical representation of the proposed method based on C-MFMPL algorithm can be performed by a set of RSS with the coordinates of a certain anchor nodes collected and stored in the buffer by each sensor node in the network which indicated according to Eq (18). Meanwhile, averaging of RSS values all over the transmitted frequencies which stored in a such buffer is expressed in Eq (19).

$$\begin{array}{c} F=\{f_{1}\left(P_{1},P_{2},P_{3}\right),f_{2}\left(P_{1},P_{2},P_{3}\right),f_{3}\left(P_{1},P_{2},P_{3}\right)\} \end{array}$$ (18)

Where

*f*_1_, *f*_2_, *f*_3_ is the transmitted frequencies.

*P*_1_, *P*_2_, *P*_3_ is the decided power levels.

$$\begin{array}{c} S_{buffer}=\{averageRSSI_{1}\left(f_{1},f_{2},f_{3}\right),averageRSS_{2}\left(f_{1},f_{2},f_{3}\right),\\ averageRSSI_{3}\left(f_{1},f_{2},f_{3}\right)\} \end{array}$$ (19)

Furthermore, Eq (20) outlines the *R*_*buffer*_ set that is collected and stored in the buffer specified by the averaging RSS readings with the coordinates of the certain anchor nodes.

$$\begin{array}{c} R_{buffer}=\left\{S_{buffer},P_{buffer}\right\} \end{array}$$ (20)

The *P*_*buffer*_ and *S*_*buffer*_ sets indicate the anchor nodes coordinates and the average RSS measurements, respectively.

The calculation of the distance (d) with respect to the averaging valid RSS values has been obtained in order to mitigate and overcome the shadow fading impacts. Moreover, to increase the accuracy of the ranging measurements specified by the distance among the sensor and each anchor node (AP), the optimization of the determined distance (d) has been applied in order to get more accurate ranging measurements which leads to obtain a better estimate of the sensor node location in the network. According to the Eq (21), the optimization of ranging distance (dOptimized) can be identified by averaging the calculated distances of the averaging valid RSS readings.

$$\begin{array}{c} d_{Optimized}=\frac{1}{3}\sum_{j=1}^{3}d_{RSSI_{j}} \end{array}$$ (21)

The major key role of determining the d_Optimized ranging mechanism is to take an advantage of not only the averaging of the RSS from one transmitted frequency, but for all the different transmitted frequencies which have different response and effects with respect to the shadow fading propagation model as well as this advantage will be reflected directly for more enhancing of the localization performance.

**Fig 6:**
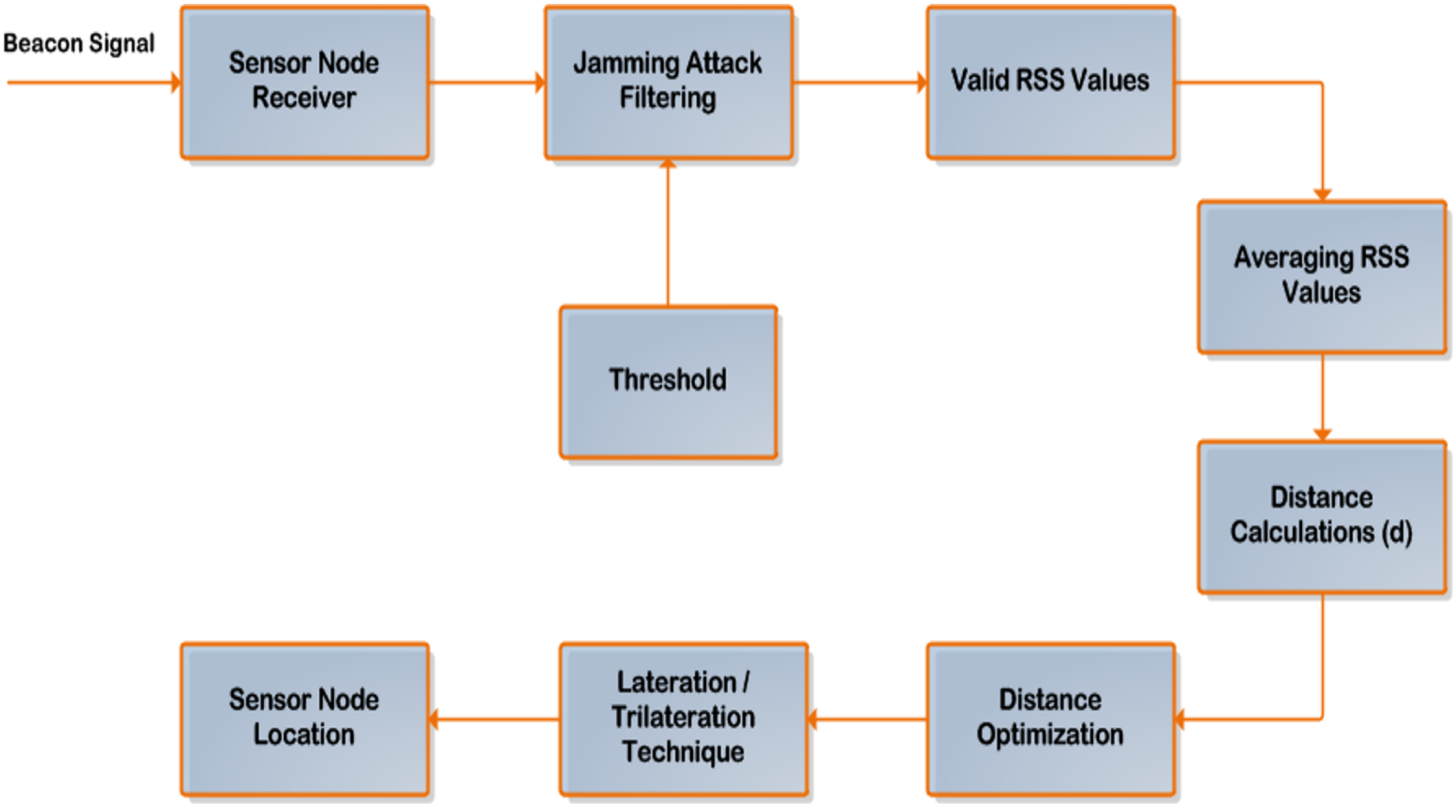
The localization strategy of the proposed method.

The point of interest which elaborate the mechanism of the proposed method localization stage based on both SF-MFMPL and C-MFMPL which has been accomplished over log normal shadow fading propagation model can be outlined regarding to Fig 6.

### Localization phase

Localization procedures include two stages which can be indicated by ranging and lateration. Ranging stage is responsible of estimating the distance d between the sensor node with respect to a certain anchor node (AP). The distance d is extracted from the RSS measurements according to Eq (22).

$$\begin{array}{c} RSS=P_{o}-10\alpha \log d \end{array}$$ (22)

Hence, the distance can be determined from Eq (23) as follows:

$$\begin{array}{c} d=10{(}^{\frac{P_{o}-RSS}{10\alpha }}) \end{array}$$ (23)

where: *P*_*o*_ is the power received in dBm at a 1-m distance; d is the distance between the node and the AP; *α* is the path loss component.

Regarding the lateration technique, the location of the sensor nodes has been obtained through measures the distance estimations with respect to various anchor nodes in the network. Two common strategies for determining the sensor nodes position can be indicated as non-linear least square (NLS) as well as linear square (LS) techniques. For NLS technique the distance estimation di and the known coordinates Li = (xi, yi) for such landmark is utilized to determine the sensor node location $\left(\hat{x},\hat{y}\right)$ according to Eq (24).

$$\begin{array}{c} \left(\hat{x},\hat{y}\right)=\arg min_{x,y}\sum_{i=1}^{n}{\left[\sqrt{{\left(xi-x\right)}^{2}+{\left(yi-y\right)}^{2}}-\hat{d_{i}}\right]}^{2} \end{array}$$ (24)

where i = 1…n for n total landmarks.

Since the computations is quite complex, an approximation solution of this equation can be obtained according to Eq (25) [23].

$$\begin{array}{c} A\hat{P}=b \end{array}$$ (25)

where:

$$\begin{array}{c} A=(\begin{array}{cc} x1-\frac{1}{n}\sum_{i=1}^{n}xi & xn-\frac{1}{n}\sum_{i=1}^{n}xi\\ y1-\frac{1}{n}\sum_{i=1}^{n}yi & yn-\frac{1}{n}\sum_{i=1}^{n}yi \end{array}) \end{array}$$ (26)

and:

$$\begin{array}{c} b=\frac{1}{2}(\begin{array}{c} \left(x_{1}^{2}-\frac{1}{n}\sum_{i=1}^{n}x_{i}^{2}\right)+\left(y_{1}^{2}-\frac{1}{n}\sum_{i=1}^{n}y_{i}^{2}\right)\\ \left(x_{n}^{2}-\frac{1}{n}\sum_{i=1}^{n}x_{i}^{2}\right)+\left(y_{1}^{2}-\frac{1}{n}\sum_{i=1}^{n}y_{i}^{2}\right)-\left({\hat{d}}_{n}^{2}-\frac{1}{n}\sum_{i=1}^{n}{\hat{d}}_{i}^{2}\right) \end{array}) \end{array}$$ (27)

A is depicted the coordinates of such landmark and b is described by the distance estimation to such landmark. Therefore, The estimation of the sensor node location can be solved regarding to Eq (28).

$$\begin{array}{c} \hat{P}={\left(A^{T}A\right)}^{-1}A^{T}b \end{array}$$ (28)

Furthermore, in light of the localization process based on trilateration strategy, the coordinates of the implemented anchor nodes is characterized by the i-th beacon Bi are (xBi, yBi), the unknown sensor node coordinates is defined by (x, y) while RSSIi and di indicates the signal strength as well as the distance estimations respectively among the sensor node and each anchor node. Hence, assuming three beacon nodes (b1, b2, b3) which defined by three estimated distances (d1, d2, d3), so that the sensor node location coordinates can be determined as follows [54, 55]:

$$\begin{array}{ccc} {\left(x-x_{B1}\right)}^{2}+{\left(y-y_{B1}\right)}^{2} & = & d_{1}^{2}\\ {\left(x-x_{B2}\right)}^{2}+{\left(y-y_{B2}\right)}^{2} & = & d_{2}^{2}\\ {\left(x-x_{B3}\right)}^{2}+{\left(y-y_{B3}\right)}^{2} & = & d_{3}^{2} \end{array}$$ (29)

By subtracting the earlier two equations from the third one, we can obtain the following equations:

$$\begin{array}{ccc} 2\left(x_{B3}-x_{B1}\right)x+2\left(y_{B3}-y_{B1}\right)y & = & x_{B3}^{2}-x_{B1}^{2}+y_{B3}^{2}-y_{B1}^{2}+d_{1}^{2}-d_{3}^{2}\\ 2\left(x_{B3}-x_{B2}\right)x+2\left(y_{B3}-y_{B2}\right)y & = & x_{B3}^{2}-x_{B2}^{2}+y_{B3}^{2}-y_{B2}^{2}+d_{2}^{2}-d_{3}^{2} \end{array}$$ (30)

By expressing Eq (30) in matrix form, we obtain:

$$\begin{array}{c} \left[\begin{array}{c} x\\ y \end{array}]={\left[\begin{array}{cc} 2(x_{B3}-x_{B1}) & 2(y_{B3}-y_{B1})\\ 2(x_{B3}-x_{B2}) & 2(y_{B3}-y_{B2}) \end{array}\right]}^{-1}\cdot [\begin{array}{c} x_{B3}^{2}-x_{B1}^{2}+y_{B3}^{2}-y_{B1}^{2}+d_{1}^{2}-d_{3}^{2}\\ x_{B3}^{2}-x_{B2}^{2}+y_{B3}^{2}-y_{B2}^{2}+d_{2}^{2}-d_{3}^{2} \end{array}\right] \end{array}$$ (31)

However, instead of utilizing real distance di, it is reasonable to use the noisy estimations $\hat{d_{i}}$, therfore Eq (31) signified as follows:

$$\begin{array}{ccc} {\left(\hat{x}-x_{B1}\right)}^{2}+{\left(\hat{y}-y_{B1}\right)}^{2} & = & {\hat{d}}_{1}^{2}\\ {\left(\hat{x}-x_{B2}\right)}^{2}+{\left(\hat{y}-y_{B2}\right)}^{2} & = & {\hat{d}}_{2}^{2}\\ {\left(\hat{x}-x_{B3}\right)}^{2}+{\left(\hat{y}-y_{B3}\right)}^{2} & = & {\hat{d}}_{3}^{2} \end{array}$$ (32)

$$\begin{array}{c} \left[\begin{array}{c} \hat{x}\\ \hat{y} \end{array}]={\left[\begin{array}{cc} 2(x_{B3}-x_{B1}) & 2(y_{B3}-y_{B1})\\ 2(x_{B3}-x_{B2}) & 2(y_{B3}-y_{B2}) \end{array}\right]}^{-1}\cdot [\begin{array}{c} x_{B3}^{2}-x_{B1}^{2}+y_{B3}^{2}-y_{B1}^{2}+{\hat{d}}_{1}^{2}-{\hat{d}}_{3}^{2}\\ x_{B3}^{2}-x_{B2}^{2}+y_{B3}^{2}-y_{B2}^{2}+{\hat{d}}_{2}^{2}-{\hat{d}}_{3}^{2} \end{array}\right] \end{array}$$ (33)

where $\left(\hat{x},\hat{y}\right)$ is the sensor node coordinate estimation. By subtracting Eq (33) from Eq (31), we can obtain the localization error of the unknown sensor node as follow:

$$\begin{array}{c} \left[\begin{array}{c} \hat{x}-x\\ \hat{y}-y \end{array}]={\left[\begin{array}{cc} 2(x_{B3}-x_{B1}) & 2(y_{B3}-y_{B1})\\ 2(x_{B3}-x_{B2}) & 2(y_{B3}-y_{B2}) \end{array}\right]}^{-1}\cdot [\begin{array}{c} \left({\hat{d}}_{1}^{2}-d_{1}^{2}\right)-\left({\hat{d}}_{3}^{2}-d_{3}^{2}\right)\\ \left({\hat{d}}_{2}^{2}-d_{2}^{2}\right)-\left({\hat{d}}_{3}^{2}-d_{3}^{2}\right) \end{array}\right] \end{array}$$ (34)

In perspective of Eq (34), it shows the localization error for using the trilateration strategy, which is related to the RSS ranging estimation deviations and as well as the coordinates of the anchor nodes. Since the anchor nodes are deployed in a fixed point with priori known and unchanged coordinates, therefore the localization error is depended directly on the RSS ranging estimation errors only. Hence, the localization error can be obtained regarding to the equation below:

$$\begin{array}{c} Localization\;\;Error\;\;\;\left(E\right)=[\begin{array}{c} \left({\hat{d}}_{1}^{2}-d_{1}^{2}\right)-\left({\hat{d}}_{3}^{2}-d_{3}^{2}\right)\\ \left({\hat{d}}_{2}^{2}-d_{2}^{2}\right)-\left({\hat{d}}_{3}^{2}-d_{3}^{2}\right) \end{array}] \end{array}$$ (35)

**Fig 7:**
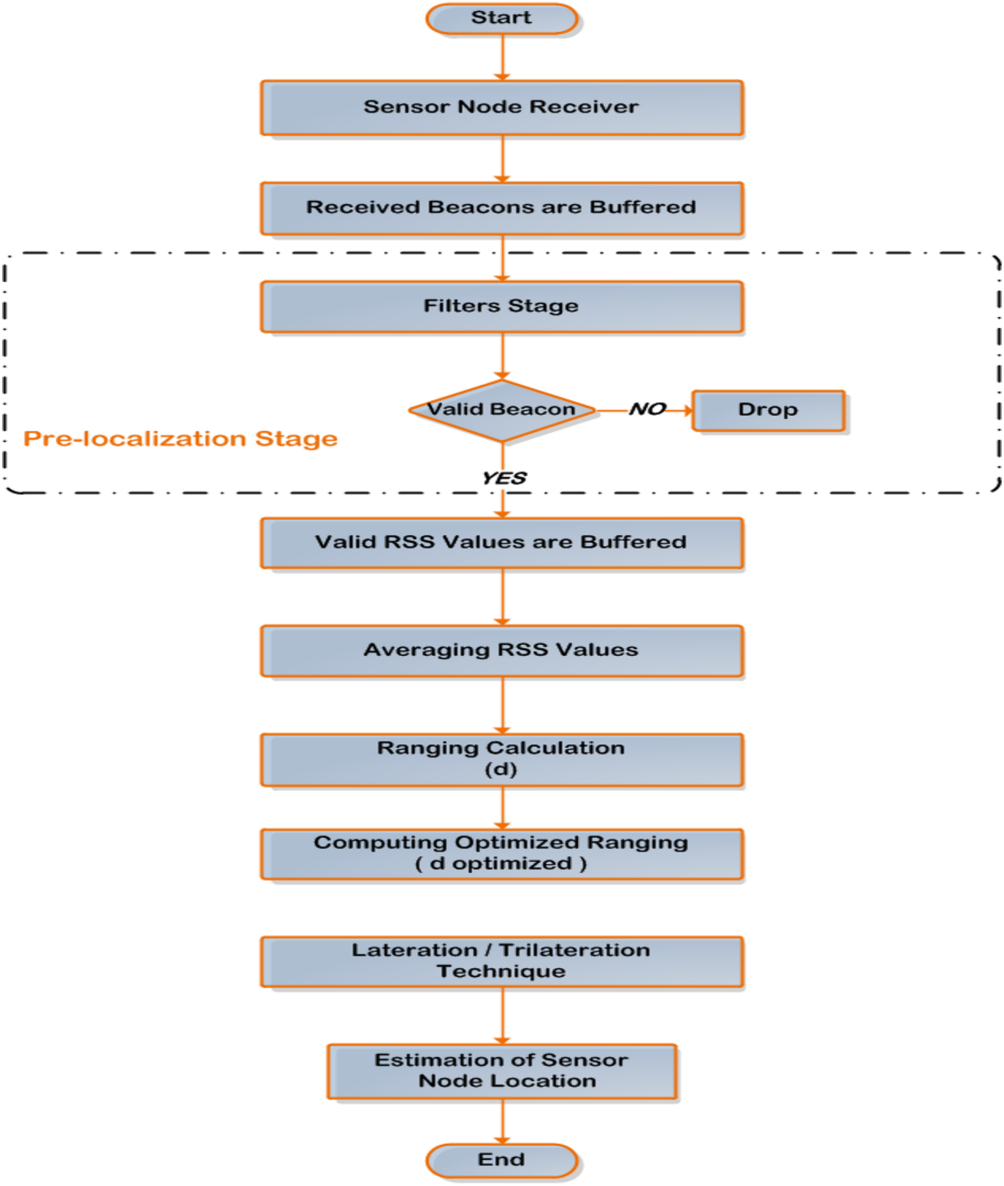
Flow chart of the proposed algorithm mechanism.

The systematic flow chart which portrays the mechanism of the proposed solution for estimating the sensor node location can be expressed according to Fig 7.

## Performance analysis and results

The simulation testing of the proposed approach has been performed by using MATLAB. The terrain network area comprises of two dimensions (1000 * 1000) meter. The number and deployment of the anchor nodes (APs) in the network is quite significant. Therefore, we utilize a few plausible number of anchor nodes deployment with 20 nodes distributed uniformly around the perimeter (5 beacon nodes at each direction). These anchor nodes are scattered in a uniform distribution over an area of 1000 * 1000 m. There are 15 multiple jammers and 100 sensor nodes deployed randomly in the network terrain. The localization must be carried out for these sensor nodes. This section introduces the effectiveness localization performance evaluation of the proposed solutions step function multiple frequency multiple power localization (SF-MFMPL) technique for finding the position of the sensor nodes for facing the log normal shadow fading propagation model, we accomplish two techniques to specify the MFMPL first is SF-MFMPL and the second technique is combination multiple frequency multiple power localization (C-MFMPL) scheme. Furthermore, to obtain efficient averaging strategy of the valid RSS readings which have considerable effect due to constant and random jammers for the case using a single set of transmitting frequencies, so that another strategy of the MFMPL algorithm has been achieved through utilizing two sets of transmitting frequencies shared between all the implemented anchor nodes in a uniformed sequence as follow:

- Even anchor nodes using set 1 frequencies (400, 600, 800) MHz for transmission.
- Odd anchor nodes using set 2 frequencies (500, 700, 900) MHz for transmission.

With a specific end goal to assess the performance of our proposed method we utilize the following simulation specifications as summarized in Table 1.

**Table 1:** Simulation specifications of the proposed system model.

System Model	Simulation Specifications
Network Area	1000 * 1000 m
Frequency Used by AP	Set 1 (400, 600, 800) MHZ
	Set 2 (500, 700, 900) MHz
Power Used by AP	4, 6, 8 dB
No of sensor	100
No. of random jammers	5
No. of Constant jammers	5
No. of capture replay jammer	5
Sensor and jammer nodes Placement	Random
Anchor nodes (Aps) Placement	Around Perimeter
Propagation Model	Log Normal Shadow Fading

In this section, the localization performance analysis has been accomplished for our proposed techniques SF-MFMPL and C-MFMPL respectively against multiple types of jamming attacks on the network as well as for facing the log normal shadow fading model in accordance to the simulation cases as follows:

Case I: path loss exponent *α* = 3, standard deviation of the log-normal shadow fading *σ* = 5 dB;

Case II: path loss exponent *α* = 4, standard deviation of the log-normal shadow fading *σ* = 6 dB.

**Fig 8:**
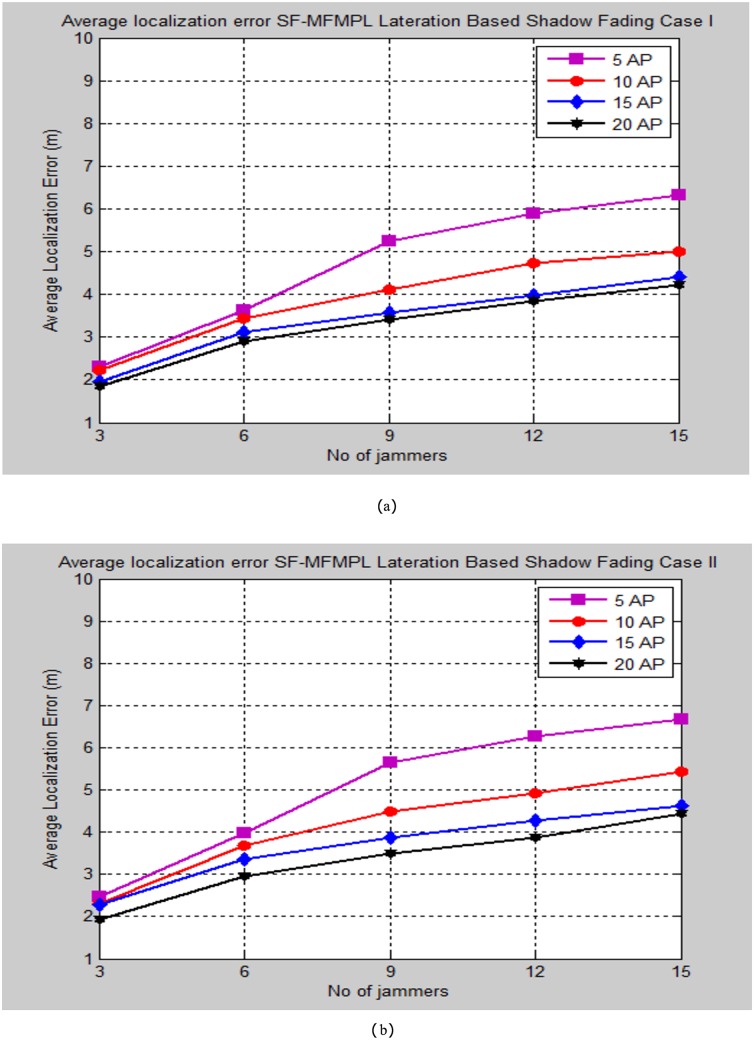
Average localization error versus number of the jammers for SF-MFMPL algorithm based on lateration technique. (a) Shadow Fading Case I; (b)Shadow Fading Case II.

**Fig 9:**
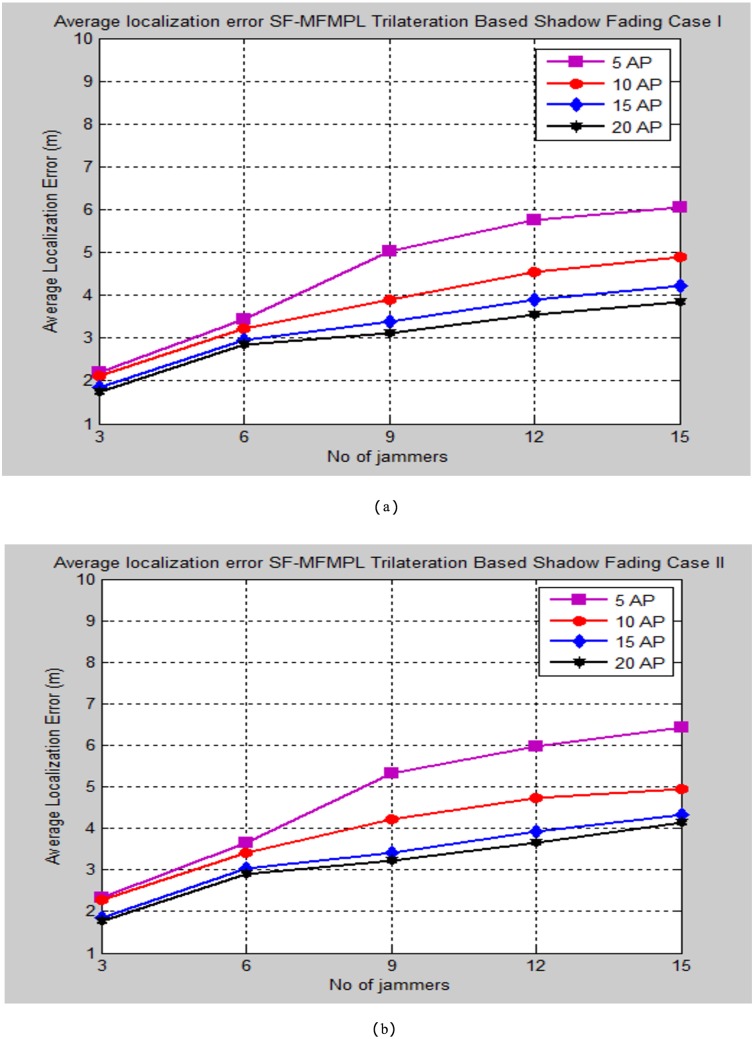
Average localization error versus number of the jammers for SF-MFMPL algorithm based on trilateration technique. (a) Shadow Fading Case I; (b) Shadow Fading Case II.

The simulation testing evaluation specified by the average localization error is determined between the estimated and real positions of the sensor nodes in the network. The performance analysis is performed for the specified propagation model cases through changing the number of multiple jammers in the simulated network as well as for different selection number of the anchor nodes. Figs 8 and 9 illustrate the average localization error of the SF-MFMPL algorithm according to the two cases of the shadow fading model as well as for lateration and trilateration techniques respectively.

It is definitely comprehended that extending the number of the anchor nodes in the network as the need to enhance the localization performance. The simulation results regarding to Figs 8 and 9 still efficient and accurate in terms of localization accuracy regarding to a few logical numbers of anchor nodes deployment configuration at the network for both of lateration and trilateration techniques as well as the effective averaging technique with the significant of different frequencies features for overcoming the shadow fading effects in the wireless network environment.

**Fig 10:**
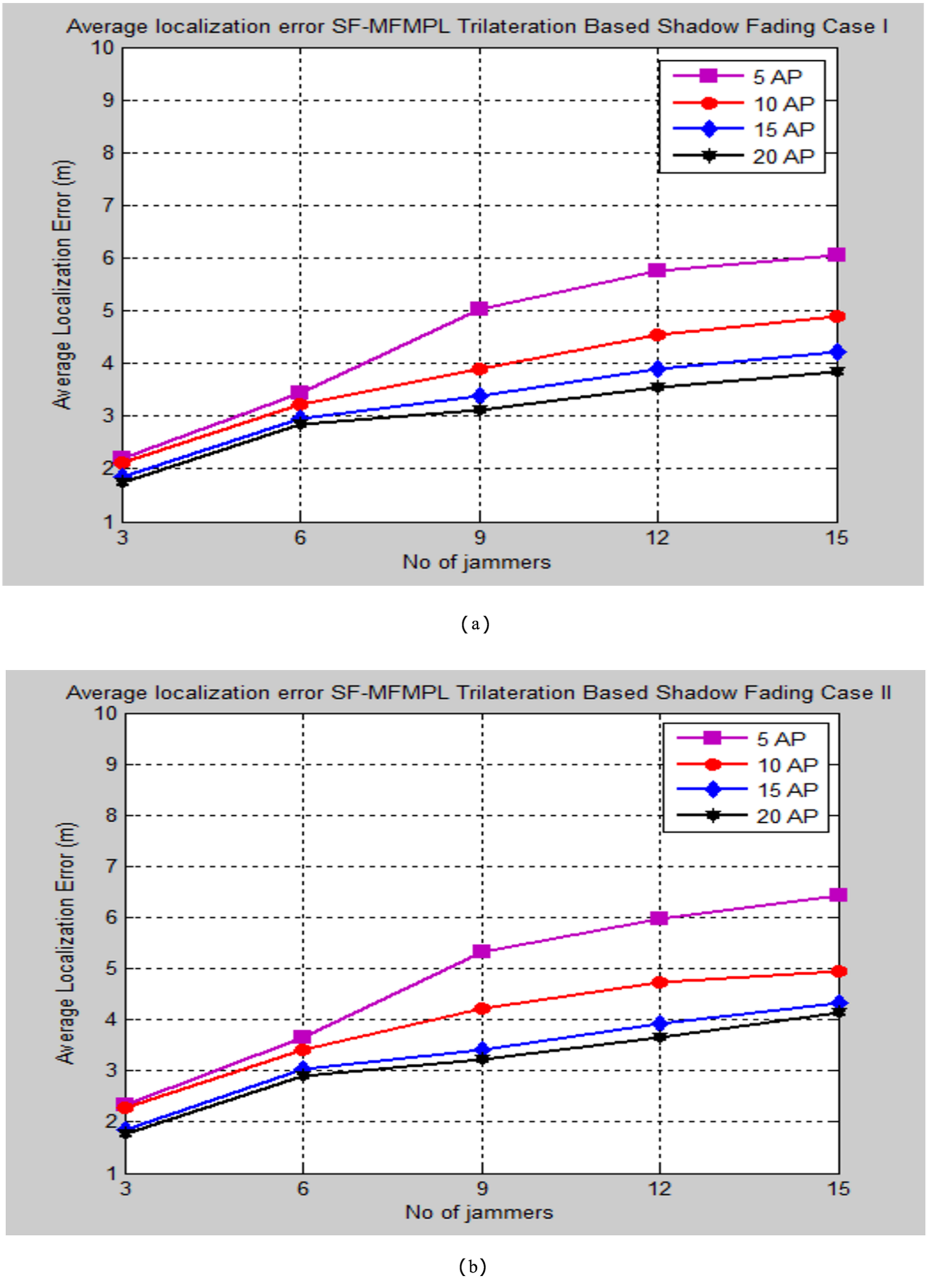
Average localization error versus number of the jammers for C-MFMPL algorithm based on lateration technique. (a) Shadow Fading Case I; (b) Shadow Fading Case II.

**Fig 11:**
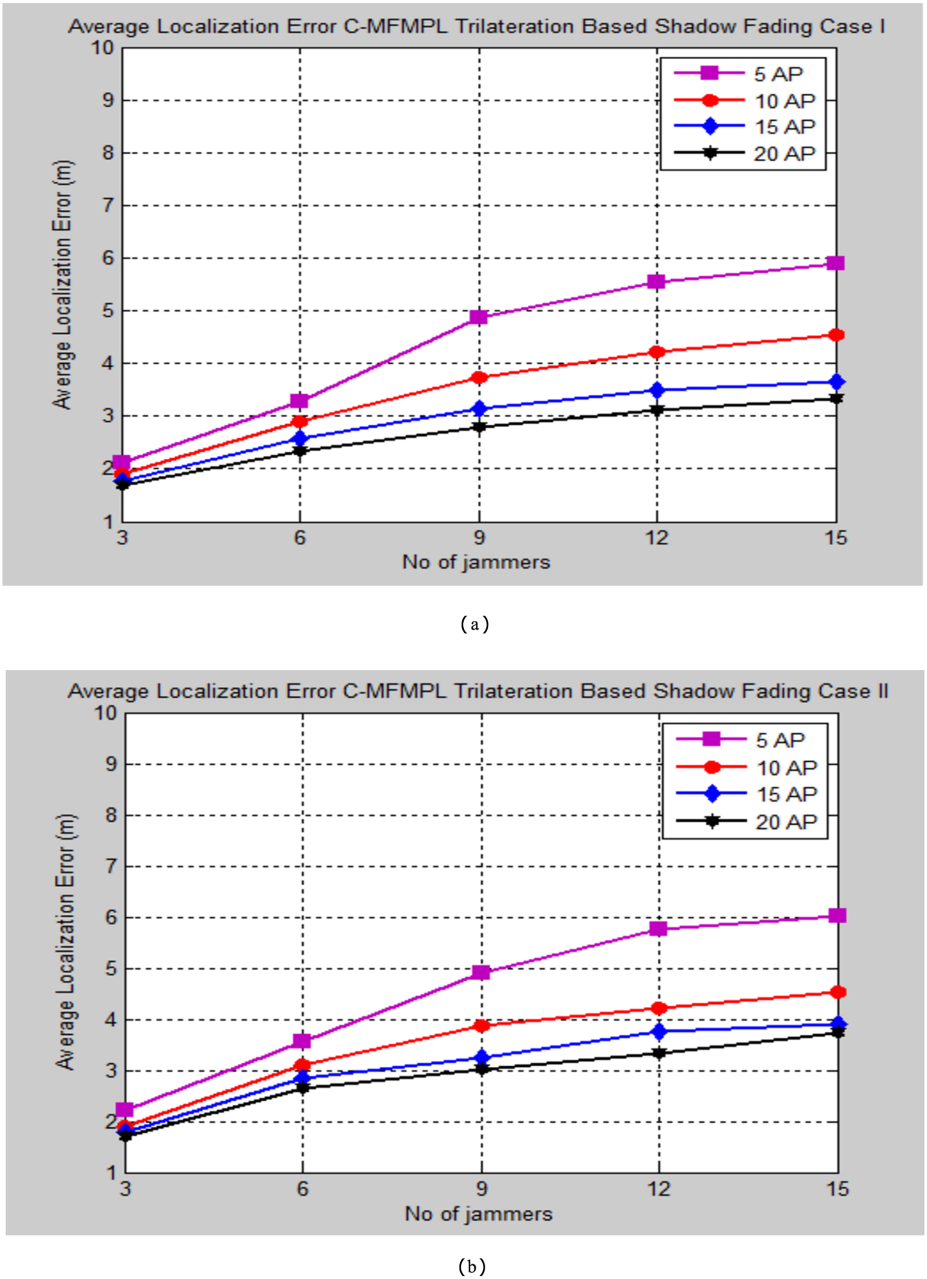
Average localization error versus number of the jammers for C-MFMPL algorithm based on trilateration technique. (a) Shadow Fading Case I; (b) Shadow Fading Case II.

Moreover, Figs 10 and 11 demonstrate the average localization error of C-MFMPL with respect to change the number of multiple jammers attack in the network and according to the lateration and trilateration techniques respectively.

In fact, the features of the averaging technique which has been used to overcome the shadow fading influence have a different strategy for both of the proposed methods. In the proposed method C-MFMPL, the averaging mechanism has been obtained through taking into consideration all the transmitted frequencies and power levels together for measuring the averaging of each RSS, while according to the other proposed method SF-MFMPL the averaging strategy of the RSS has been achieved through number of transmitted beacon signals with respect to the same assigned power levels and frequencies. Definitely, the characterizations of log normal shadow fading parameters denoted by (*α*, *σ*) are subjected and depended directly on the frequency and the power level which have been used. Therefore, different frequencies and power levels have different effects according to the shadow fading characterizations in the wireless networks. This is a reflection of the robustness of our proposed algorithm in the face of the shadow fading with an accurate localization performance as be shown in the simulation results above.

**Fig 12:**
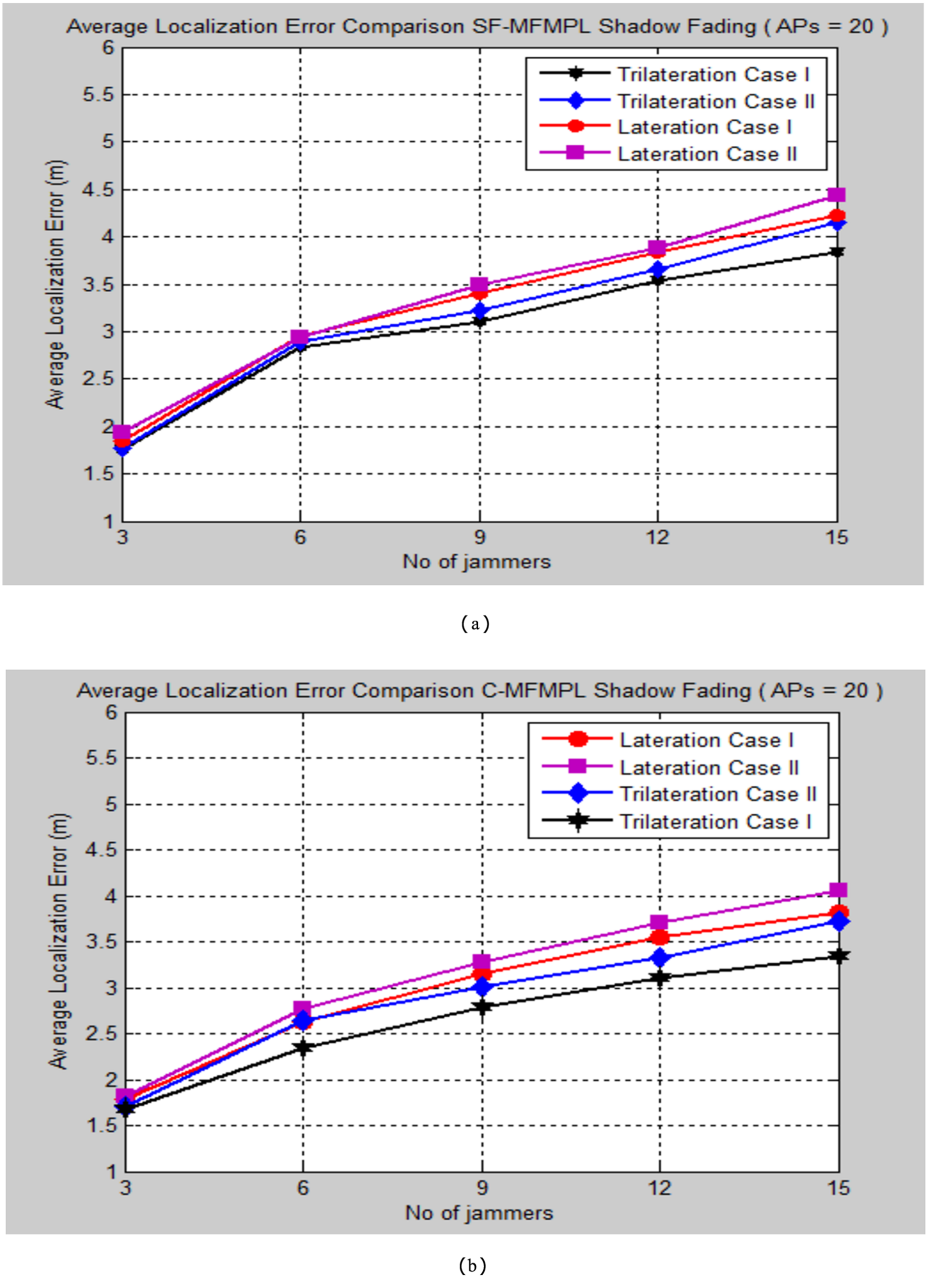
A comparison of the average localization error for SF-MFMPL and C-MFMPL algoritms based on lateration and trilateration over the two implemented cases of the shadow fading model. (a) SF-MFMPL Strategy; (b) C-MFMPL Strategy.

A comparison of the localization performance between the SF-MFMPL and C-MFMPL algorithms as indicated by 20 anchor nodes (APs) which are deployed in the network as well as for the two propagation model cases is illustrated in Fig 12.

Regarding the simulation results as illustrated in Fig 12, the localization performance of the C-MFMPL approach showed more enhancements in the average localization error as compared with the SF-MFMPL method. In fact, the different strategies which have been achieved for indicating the multiple frequency and power level mechanism for our proposed algorithms will drive to different simulation results especially for facing the shadow fading impacts. Besides, the simulation results proved that the performance of trilateration technique is better than the lateration method for the two proposed algorithms and for both cases of the propagation model as well. Table 2 outlines the comparison synopsis of the localization performance evaluation of the two proposed techniques as indicated by 20 anchor nodes in the network for the two cases of the propagation model.

**Table 2:** Average localization error comparison of the two proposed solutions according to 20 anchors (APs), 15 numbers of multiple jammers and two propagation model cases.

Propagation Model Case	Average Localization Error (m)		Average Localization Error (m)	
	SF-MFMPL		C-MFMPL	
	Lateration	Trilateration	Lateration	Trilateration
**Case I**	4.23	3.84	3.82	3.34
**Case II**	4.43	4.15	4.05	3.72

**Fig 13:**
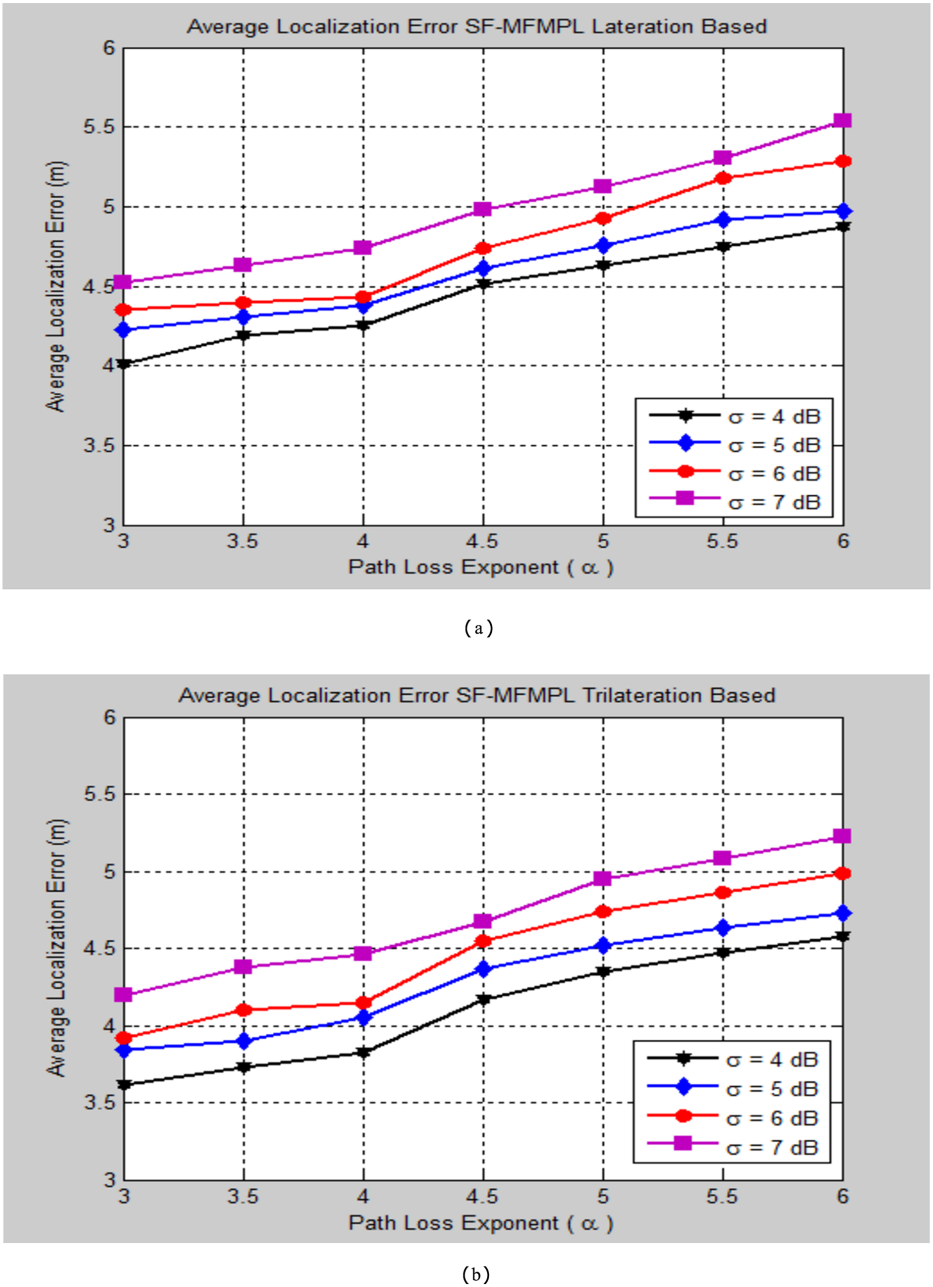
Average localization error versus different path loss exponent values for the SF-MFMPL algorithm (No. of multiple jammers = 15, APs = 20). (a) Lateration Based; (b) Trilateration Based.

**Fig 14:**
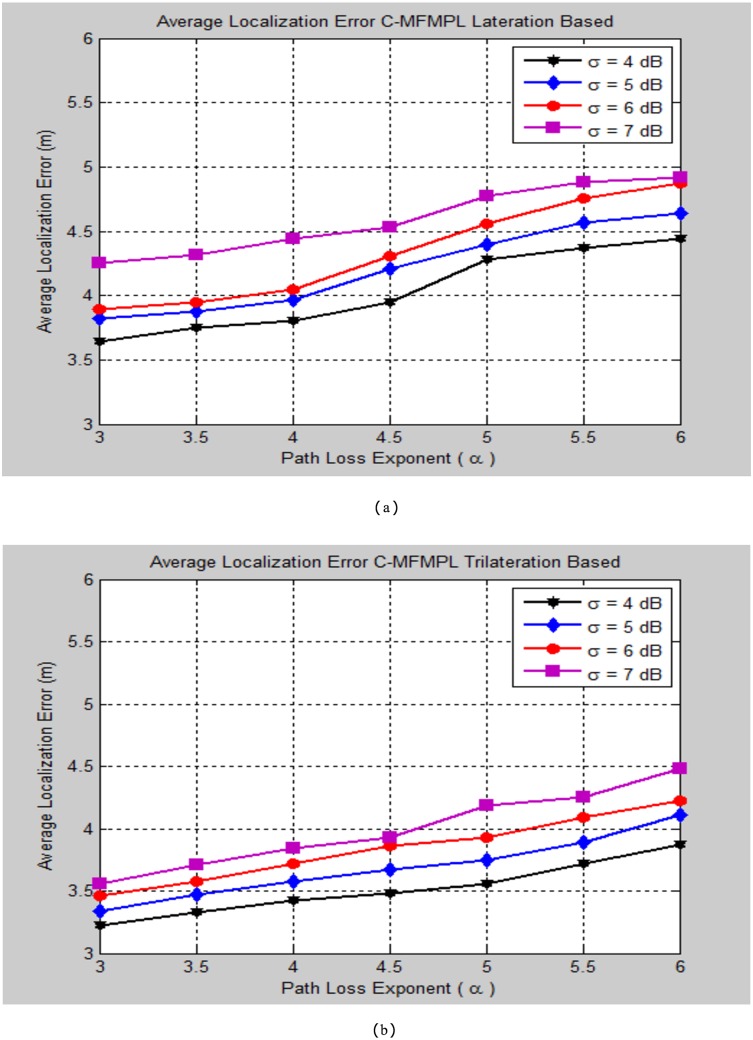
Average localization error versus different path loss exponent values for the C-MFMPL algorithm (No. of multiple jammers = 15, APs = 20). (a) Lateration Based; (b) Trilateration Based.

The impact of varying the path loss component (*α*) on the localization performance concerning to various standard deviation (*σ*) of the log normal shadow fading model has been taken into the simulation testing consideration of the proposed algorithms SF-MFMPL and C-MFMPL respectively. Figs 13 and 14 outline the localization performance under these impacts.

In practical environment, the parameters of the log normal shadow fading are vulnerable to error as well as unpredictable. For this reason the simulation tests of the proposed algorithms have been accomplished in order to take into account the impacts of varying these parameters on the localization performance evaluation. As represented in the simulation results of Figs 13 and 14, the proposed algorithm demonstrated an accurate and effective localization performance for estimating the sensor node location with a reasonable localization error rates. Furthermore, the simulation results proved that the performance of trilateration strategy is superior to the lateration technique for the two proposed algorithms. Tables 3 and 4 outline the comparison synopsis of the localization performance evaluation of our proposed techniques indicated by varying the shadow fading parameters with respect to the lateration and trilateration strategies respectively.

**Table 3:** Average localization error comparison of the two proposed solutions according to changing the shadow fading propagation parameters (lateration based).

Path Loss Exponent (*α*)	Average Localization Error (m)							
	SF-MFMPL Lateration Based				C-MFMPL Lateration Based			
	*σ* = 4	*σ* = 5	*σ* = 6	*σ* = 7	*σ* = 4	*σ* = 5	*σ* = 6	*σ* = 7
***α* = 3**	***4.01***	***4.23***	***4.35***	***4.52***	***3.64***	***3.82***	***3.89***	***4.25***
***α* = 4**	***4.25***	***4.38***	***4.43***	***4.74***	***3.80***	***3.97***	***4.05***	***4.44***
***α* = 5**	***4.63***	***4.76***	***4.93***	***5.12***	***4.28***	***4.40***	***4.56***	***4.78***
***α* = 6**	***4.87***	***4.97***	***5.28***	***5.54***	***4.44***	***4.64***	***4.87***	***4.92***

**Table 4:** Average localization error comparison of the two proposed solutions according to changing the shadow fading propagation parameters (Trilateration based).

Path Loss Exponent(*α*)	Average Localization Error (m)							
	SF-MFMPL Trilateration Based				C-MFMPL Trilateration Based			
	*σ* = 4	*σ* = 5	*σ* = 6	*σ* = 7	*σ* = 4	*σ* = 5	*σ* = 6	*σ* = 7
***α* = 3**	***3.61***	***3.84***	***3.92***	***4.20***	***3.22***	***3.34***	***3.45***	***3.56***
***α* = 4**	***3.82***	***4.05***	***4.15***	***4.46***	***3.41***	***3.58***	***3.72***	***3.83***
***α* = 5**	***4.35***	***4.52***	***4.74***	***4.95***	***3.55***	***3.74***	***3.91***	***4.19***
***α* = 6**	***4.58***	***4.73***	***4.99***	***5.22***	***3.86***	***4.11***	***4.22***	***4.48***

In this paper, we discuss the wide range capability of the received signal strength localization based over the existence of multiple jammers influence. An exploration of the localization performance response to unexpected three different numbers of jammers exists in the network at the same time has been accomplished. Hence, a comparison of the simulation results has been obtained according to the same specifications as well as the system model implementation of the proposed modality for various required scenarios. In addition, taking into consideration the performance evaluation of the ideal case which can be denoted as the localization performance of the proposed system in the absence of jamming attack impacts. The comparison results are performed in order to show the robustness of our approach as well as the effectiveness of the jamming attack interference on the localization performance based on signal strength.

**Fig 15:**
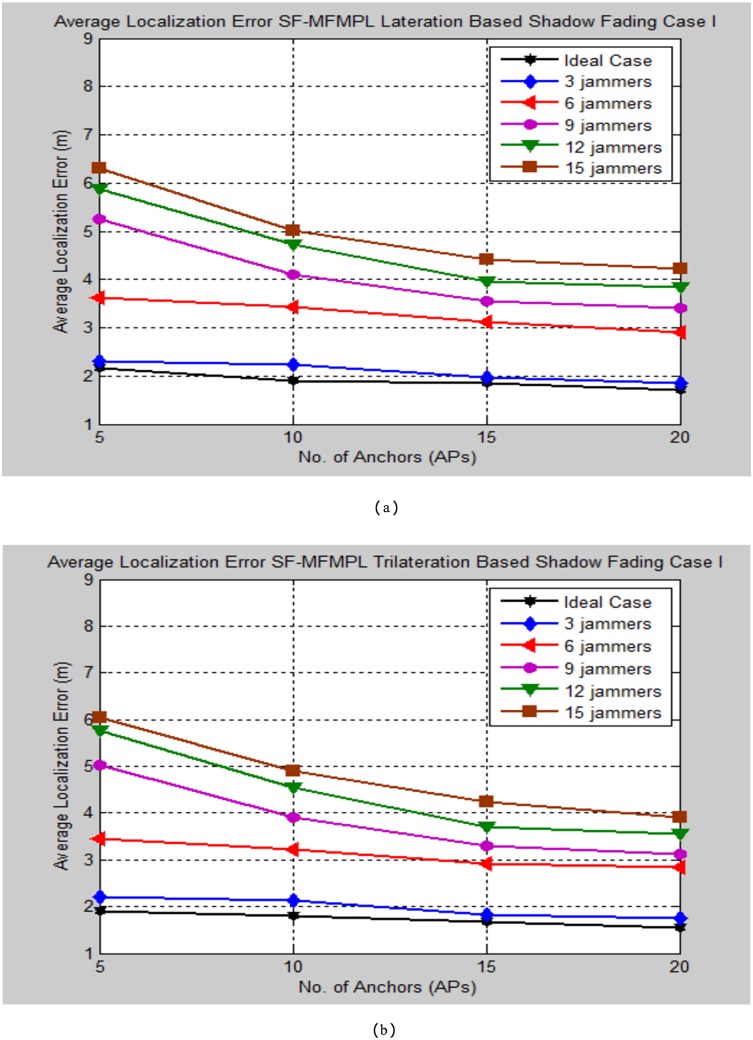
Average localization error comparison for the implemented cases of the SF-MFMPL under log normal shadow fading case I. (a) Lateration Based; (b) Trilateration Based.

**Fig 16:**
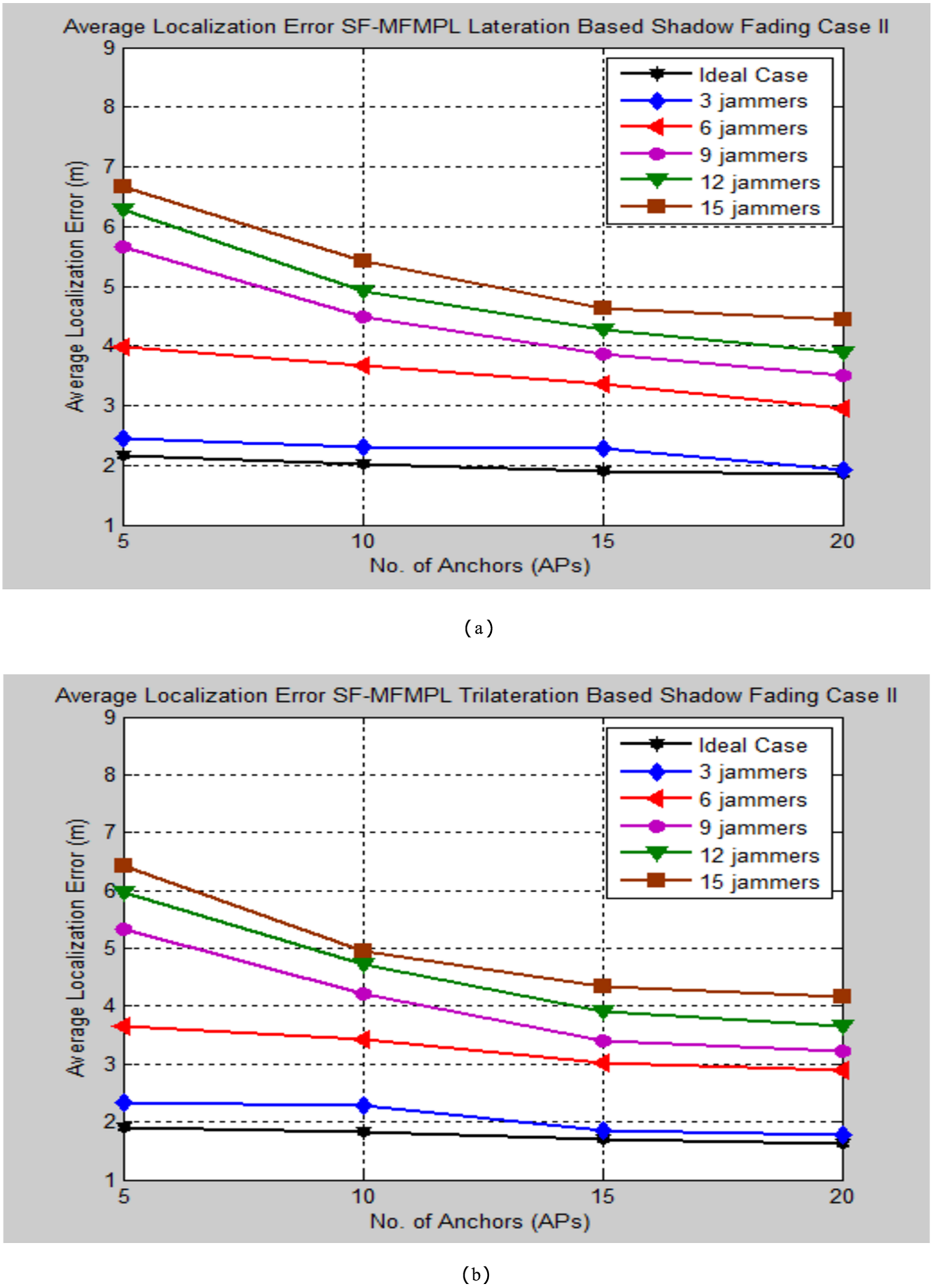
Average localization error comparison for the implemented cases of the SF-MFMPL under log normal shadow fading case II. (a) Lateration Based; (b) Trilateration Based.

Figs 15 and 16 outline a comparison of the average localization error between the ideal case and different number of multiple jammers in the network for the SF-MFMPL under a shadow fading channel propagation model which is specified by the lateration and trilateration method respectively.

According to the simulation results as outlined in Figs 15 and 16, the performance evaluation of the proposed system in the case of three numbers of jammers exists in the network showed a very close average localization error as compared with the ideal case and the localization error increased slightly with respect to increasing the number of jammers. Meanwhile, the localization performance of the proposed system as the number of jammers increased to 15 is very accurate and have a powerful performance specially for range based received signal indicator localization based sensor node location in the presence of a harsh jamming environment. Tables 5–8 summarize the tested simulation results comparison for SF-MFMPL approach.

**Table 5:** Comparison of average localization error (m) of SF-MFMPL algorithm lateration based shadow fading model case I.

*Average localization error (m) SF-MFMPL Shadow Fading Lateration Based Case I*						
*No. of Aps*	*Ideal Case*	*3 Jammers*	*6 Jammers*	*9 Jammers*	*12 Jammers*	*15 Jammers*
***5***	*2.15*	*2.30*	*3.62*	*5.25*	*5.88*	*6.31*
***10***	*1.91*	*2.23*	*3.44*	*4.10*	*4.73*	*5.01*
***15***	*1.84*	*1.97*	*3.11*	*3.56*	*3.97*	*4.41*
***20***	*1.70*	*1.84*	*2.91*	*3.41*	*3.84*	*4.23*

**Table 6:** Comparison of average localization error (m) of SF-MFMPL algorithm trilateration based shadow fading model case I.

*Average localization error (m) SF-MFMPL Shadow Fading Trilateration Based Case I*						
*No. of Aps*	*Ideal Case*	*3 Jammers*	*6 Jammers*	*9 Jammers*	*12 Jammers*	*15 Jammers*
***5***	*1.89*	*2.20*	*3.44*	*5.03*	*5.76*	*6.05*
***10***	*1.80*	*2.12*	*3.21*	*3.91*	*4.54*	*4.89*
***15***	*1.68*	*1.82*	*2.96*	*3.38*	*3.89*	*4.21*
***20***	*1.54*	*1.75*	*2.84*	*3.11*	*3.54*	*3.84*

**Table 7:** Comparison of average localization error (m) of SF-MFMPL algorithm lateration based shadow fading model case II.

*Average localization error (m) SF-MFMPL Shadow Fading Lateration Based Case II*						
*No. of APs*	*Ideal Case*	*3 Jammers*	*6 Jammers*	*9 Jammers*	*12 Jammers*	*15 Jammers*
***5***	*2.17*	*2.46*	*3.98*	*5.66*	*6.28*	*6.67*
***10***	*2.02*	*2.31*	*3.67*	*4.48*	*4.92*	*5.42*
***15***	*1.89*	*2.28*	*3.37*	*3.86*	*4.27*	*4.62*
***20***	*1.84*	*1.93*	*2.96*	*3.50*	*3.88*	*4.43*

**Table 8:** Comparison of average localization error (m) of SF-MFMPL algorithm trilateration based shadow fading model case II.

*Average localization error (m) SF-MFMPL Shadow Fading Trilateration Based Case II*						
*No. of APs*	*Ideal Case*	*3 Jammers*	*6 Jammers*	*9 Jammers*	*12 Jammers*	*15 Jammers*
***5***	*1.91*	*2.34*	*3.65*	*5.33*	*5.97*	*6.43*
***10***	*1.82*	*2.28*	*3.42*	*4.21*	*4.73*	*4.96*
***15***	*1.71*	*1.86*	*3.03*	*3.41*	*3.92*	*4.34*
***20***	*1.63*	*1.77*	*2.89*	*3.23*	*3.66*	*4.15*

**Fig 17:**
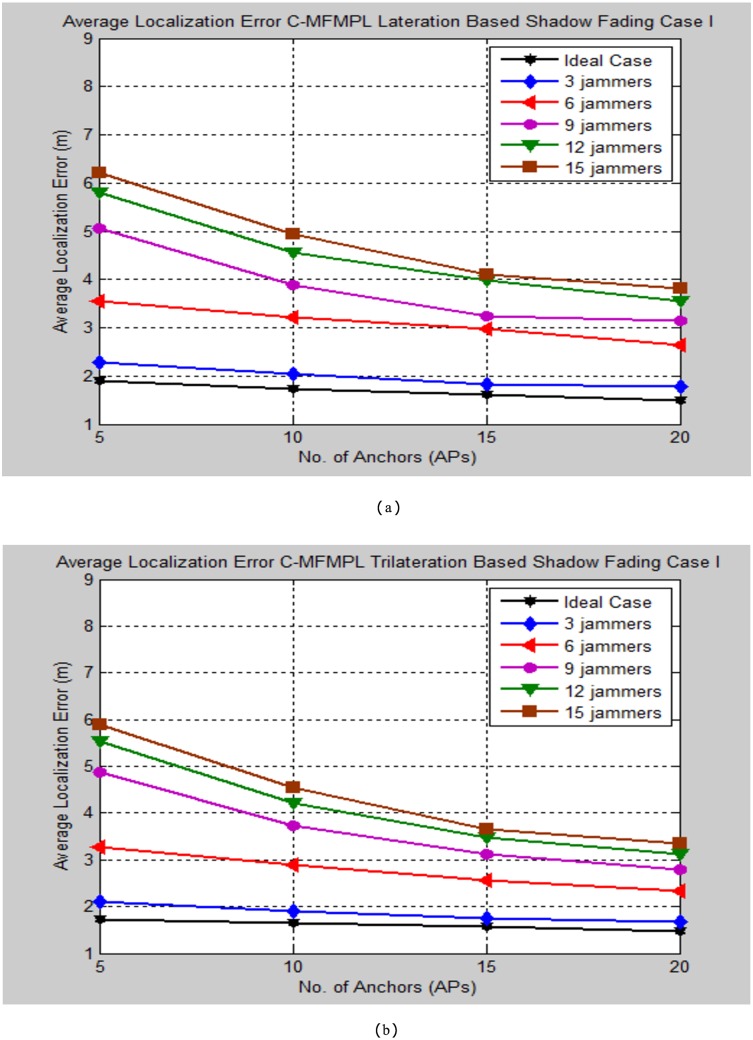
Average localization error comparison for the implemented cases of the C-MFMPL under log normal shadow fading case I. (a) Lateration Based; (b) Trilateration Based.

**Fig 18:**
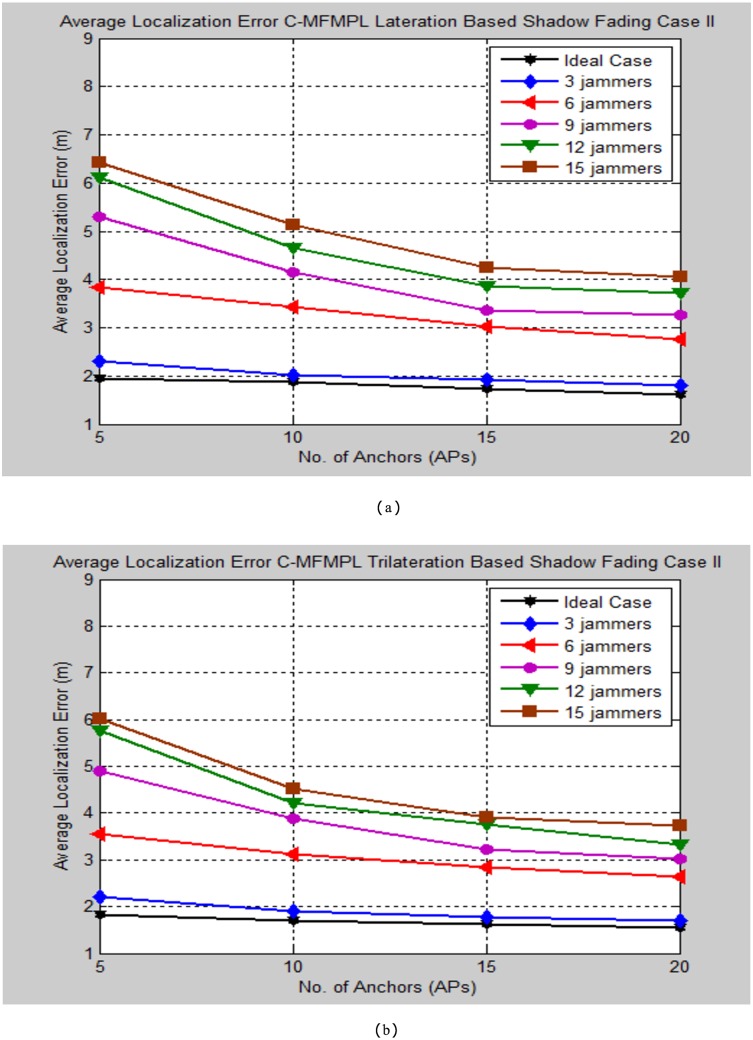
Average localization error comparison for the implemented cases of the C-MFMPL under log normal shadow fading case II. (a) Lateration Based; (b) Trilateration Based.

Another comparison of the average localization error for the implemented cases of the C-MFMPL under the two cases of the log normal shadow fading model is shown in Figs 17 and 18.

In light of the simulation results of Figs 17 and 18, it is noticed that the performance evaluation of the ideal case is very close to the proposed approach in the presence of only three multiple jammers which implemented in the network. However, the average localization error increased slightly as indicated to the other cases of increasing the number of jammers, this is normal for the reason of the amount of the beacons which have been lost due to constant and random jammers influence. Furthermore, the main observation of the performance analysis for the proposed method C-MFMPL is obtained better localization accuracy as compared with the proposed method SF-MFMPL according to all simulation testing conditions as well as for lateration and trilateration strategies. This fact can be elaborated due to the important features of the C-MFMPL for taking the great advantages of all multiple transmitted frequencies and power levels in the averaging process so as the optimized ranging in facing the shadow fading environment. Tables 9–12 illustrate the simulation testing results summarization of the C-MFMPL over different tested conditions also for lateration and trilateration as well.

**Table 9:** Comparison of average localization error (m) of C-MFMPL algorithm lateration based shadow fading model case I.

*Average localization error (m) C-MFMPL Shadow Fading Lateration Based Case I*						
*No. of APs*	*Ideal Case*	*3 Jammers*	*6 Jammers*	*9 Jammers*	*12 Jammers*	*15 Jammers*
***5***	*1.89*	*2.27*	*3.54*	*5.05*	*5.80*	*6.20*
***10***	*1.74*	*2.04*	*3.21*	*3.88*	*4.56*	*4.93*
***15***	*1.61*	*1.83*	*2.98*	*3.23*	*3.98*	*4.11*
***20***	*1.50*	*1.78*	*2.63*	*3.15*	*3.54*	*3.82*

**Table 10:** Comparison of average localization error (m) of C-MFMPL algorithm trilateration based shadow fading model case I.

*Average localization error (m) C-MFMPL Shadow Fading Trilateration Based Case I*						
*No. of APs*	*Ideal Case*	*3 Jammers*	*6 Jammers*	*9 Jammers*	*12 Jammers*	*15 Jammers*
***5***	*1.73*	*2.11*	*3.28*	*4.88*	*5.54*	*5.89*
***10***	*1.65*	*1.90*	*2.89*	*3.74*	*4.22*	*4.55*
***15***	*1.56*	*1.76*	*2.57*	*3.13*	*3.48*	*3.66*
***20***	*1.48*	*1.68*	*2.34*	*2.78*	*3.11*	*3.34*

**Table 11:** Comparison of average localization error (m) of C-MFMPL algorithm lateration based shadow fading model case II.

*Average localization error (m) C-MFMPL Shadow Fading Lateration Based Case II*						
*No. of APs*	*Ideal Case*	*3 Jammers*	*6 Jammers*	*9 Jammers*	*12 Jammers*	*15 Jammers*
***5***	*1.95*	*2.31*	*3.84*	*5.29*	*6.11*	*6.43*
***10***	*1.88*	*2.02*	*3.42*	*4.15*	*4.66*	*5.13*
***15***	*1.73*	*1.92*	*3.02*	*3.36*	*3.86*	*4.24*
***20***	*1.62*	*1.81*	*2.77*	*3.27*	*3.71*	*4.05*

**Table 12:** Comparison of average localization error (m) of C-MFMPL algorithm trilateration based shadow fading model case II.

*Average localization error (m) C-MFMPL Shadow Fading Trilateration Based Case II*						
*No. of APs*	*Ideal Case*	*3 Jammers*	*6 Jammers*	*9 Jammers*	*12 Jammers*	*15 Jammers*
***5***	*1.83*	*2.21*	*3.55*	*4.89*	*5.77*	*6.01*
***10***	*1.71*	*1.90*	*3.11*	*3.87*	*4.22*	*4.52*
***15***	*1.62*	*1.78*	*2.84*	*3.23*	*3.75*	*3.90*
***20***	*1.54*	*1.70*	*2.64*	*3.01*	*3.33*	*3.72*

For a general analysis of the proposed algorithms which specified by SF-MFMPL and C-MFMPL algorithm under a multiple number of jammers exist in the network, the constant and random jammers are using different frequencies for their attacking, besides our proposed approach depends on the averaging technique of the RSS over all transmitted frequencies. Therefore, a serious problem appears for applying the averaging process if the anchor nodes send beacons utilizing one set only such as (400, 600, 800)MHz, so that a new mechanism has been achieved in our proposed method by using two sets of frequencies shared between the implemented anchor nosed with uniformed sequence instead of one set only in order to mitigate and overcome these two jammers impacts. In addition, this mechanism still simple for implementation and does not need for extra hardware or increasing the complexity of the system design, while it can be achieved using multiband antenna array for the required designated two frequency sets.

However, it is possible to utilize another mechanism of transmitting beacon signals by independent sets of frequencies for each anchor node in the network, for sure this strategy will enhance the localization performance with respect to increasing the system design complexity. Meanwhile, the localization performance of our proposed method showed an effective and efficient performance enough for going for this complicated design.

Furthermore, there are even various probabilities of the jammer strategies for attacking the network by choosing the frequency which has indicated to be targeted. In this study, which is based on transmitting beacons by using the main set of frequencies (400, 600, 800) MHz, so that these targeting frequencies will be taken into consideration for this study to be attacked by a constant and random jammers. In the end, these probabilities cannot be considered as a big problem to be defeated by our proposed algorithm in any way due to the powerful design of the proposed approach.

A comparison of the proposed methods performance with the state of art indoor localization based on multi frequencies and powers [23] has been achieved. The main contribution of this work is to improve the RSS localization performance over a multipath environments by varying the transmitter’s signal power and frequency under ideal case (without attacking). Regarding the lateration based algorithm, the linear least square (LLS) average localization error is about (1.52 m) using constrained landmark with residual metric, while the average localization error is around (2.13 m) for the case of matching characteristics with deviation metric.

According to the simulation results of our proposed SF-MFMPL strategy based on lateration technique, the average localization error is around (2.15 m) in case of 5 implemented anchor nodes and it will be decreased to (1.70 m) for 20 implemented anchor nodes for shadow fading propagation model case I. Meanwhile, the average localization error is about (2.17 m) and decreased to (1.84 m) for the 5 and 20 implemented anchor nodes respectively at the shadow fading propagation model case II. On the other hand, the average localization error of our proposed method C-MFMPL based on lateration technique is about (1.89 m) in case of 5 implemented anchor nodes and it will be decreased to (1.5 m) for 20 implemented anchor nodes for shadow fading propagation model case I. Besides, the average localization error is around (1.95 m) and decreased to (1.62 m) for the 5 and 20 implemented anchor nodes respectively at the shadow fading propagation model case II. As compared to the state of art relevant work [23], the performance analysis of our proposed strategies emphasized a robust and effective localization accuracy for a large scale wireless sensor network over a shadow fading channel propagation model. Furthermore, the simulation results of our proposed schemes based on trilateration technique showed more accurate localization accuracy as compared to the lateration based and for both shadow fading propagation model implemented cases.

**Fig 19:**
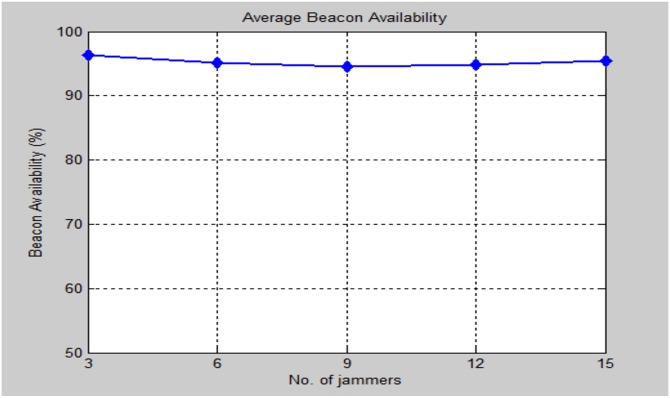
Average beacon signals availability in the presence of multiple jammers.

The standout amongst the most important criteria of the performance evaluation is the beacon signals availability at each sensor node in the presence of multiple jammers in the network. Fig 19 illustrates the effect of increasing the number of jammers and measure the availability of beacons considered in the localization process.

**Fig 20:**
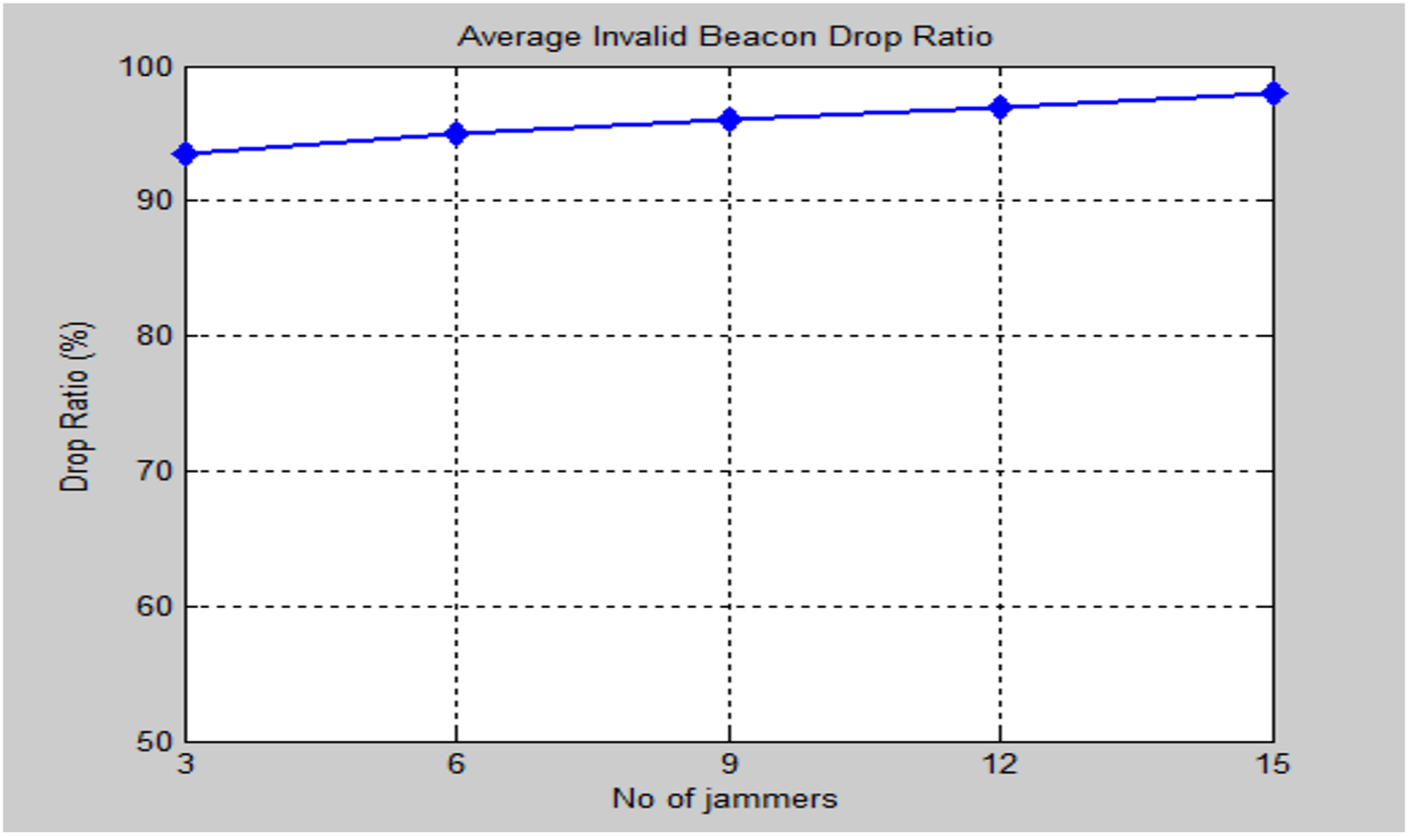
Average invalid beacons drop ratio.

In light of the simulation results of Fig 19, the observation emphasized that while increasing the number of jammers in the network with respect to the number of sensor nodes for which beacons are available for localization in our approach it is almost constant. Despite constant and random jammers is interfered the communication and cut the communication among the sensors with each anchor node through their different jamming frequencies but still the beacon availability is very high. However, these high availability of beacon signals are not all considered to be valid beacons because of some of these beacons are not actual and real ones which have been received from the anchor nodes, some of these available beacons are replayed by the capture and replay jammers. Therefore, Fig 20 shows another significant metric to describe the robustness of our proposed method which is identified by the effective percentage of dropping the invalid beacon packets. This accuracy is determined in terms of drop ratio by increasing the number of jammers in the network.

In accordance with the simulation results of Fig 20, it is noticed that as the numbers of capture and replay jammers increased in the network more invalid beacons will show up, while the effective drop ratio is very high in our approach which contributing to lesser localization error.

## Conclusion

This paper presents a powerful range based received signal strength indicator localization approach in the vicinity of multiple kinds of jammers over a log normal shadow fading environment. The proposed schemes has been accomplished using two strategies of multiple frequencies and power levels specified by SF-MFMPL and C-MFMPL respectively, including lateration and trilateration techniques, with two transmitted sets of frequencies which have been distributed uniformly among the implemented anchor nodes in order to cope with the constant and random jammers. Meanwhile, to obtain an accurate estimation of the localization performance, three stages of filters have been applied to filter out the received beacon signals with a view to overcome the impacts of the capture and replay jammers. Furthermore, the averaging of the RSS measurements all over the multiple frequencies and power levels of the proposed algorithm supported by the distance ranging optimization play the significant key role in facing the shadow fading effects. The promising results of our investigation on the proposed algorithms demonstrated a superior localization performance through the use of a few and reasonable number of anchor nodes in the network. Furthermore, the deployment configuration of the anchor nodes specified around the perimeter obtained a successful localization accuracy. In addition, the simulation results revealed that the localization performance of the proposed algorithm identified by the C-MFMPL has better performance when compared with SF-MFMPL algorithm for both lateration and trilateration techniques under the shadow fading propagation model. Finally, the justification of the system design based on our proposed methodology in face of the harsh shadow fading environment over a multiple kinds of jammers conferred a powerful and accurate localization performance. It also maintains the system design simplicity without any other complicated possibilities for the localization procedures, detection and elimination of the jammers impacts in the wireless sensor networks.
